# HCMV carriage in the elderly diminishes anti-viral functionality of the adaptive immune response resulting in virus replication at peripheral sites

**DOI:** 10.3389/fimmu.2022.1083230

**Published:** 2022-12-15

**Authors:** Emma L. Davies, Mahlaqua Noor, Eleanor Y. Lim, Charlotte J. Houldcroft, Georgina Okecha, Claire Atkinson, Matthew B. Reeves, Sarah E. Jackson, Mark R. Wills

**Affiliations:** ^1^ Department of Medicine, Cambridge Institute of Therapeutic Immunology and Infectious Disease, University of Cambridge School of Clinical Medicine, Cambridge, United Kingdom; ^2^ Institute of Immunity and Transplantation, Division of Infection and Immunity, University College London, London, United Kingdom

**Keywords:** human cytomegalovirus (HCMV), immune senescence, anti-viral T cells, aging, neutralizing antibodies, anti-viral assays, latent infection

## Abstract

Human cytomegalovirus (HCMV) infection and periodic reactivation is, generally, well controlled by adaptative immune responses in the healthy. In older people, overt HCMV disease is rarely seen despite the association of HCMV with increased risk of mortality; evidence from studies of unwell aged populations suggest that HCMV seropositivity is an important co-morbidity factor. HCMV genomes have been detected in urine from older donors, suggesting that the immune response prevents systemic disease but possibly immunomodulation due to lifelong viral carriage may alter its efficacy at peripheral tissue sites. Previously we have demonstrated that there were no age-related expansions of T cell responses to HCMV or increase in latent viral carriage with age and these T cells produced anti-viral cytokines and viremia was very rarely detected. To investigate the efficacy of anti-HCMV responses with increasing age, we used an *in vitro* Viral Dissemination Assay (VDA) using autologous dermal fibroblasts to determine the anti-viral effector capacity of total PBMC, as well as important subsets (T cells, NK cells). In parallel we assessed components of the humoral response (antibody neutralization) and combined this with qPCR detection of HCMV in blood, saliva and urine in a cohort of young and old donors. Consistent with previous studies, we again show HCMV specific cIL-10, IFNγ and TNFα T cell responses to peptides did not show an age-related defect. However, assessment of direct anti-viral cellular and antibody-mediated adaptive immune responses using the VDA shows that older donors are significantly less able to control viral dissemination in an *in vitro* assay compared to young donors. Corroborating this observation, we detected viral genomes in saliva samples only from older donors, these donors had a defect in cellular control of viral spread in our *in vitro* assay. Phenotyping of fibroblasts used in this study shows expression of a number of checkpoint inhibitor ligands which may contribute to the defects observed. The potential to therapeutically intervene in checkpoint inhibitor pathways to prevent HCMV reactivation in the unwell aged is an exciting avenue to explore.

## 1 Introduction

Susceptibility to new infections, malignancies and autoimmune diseases with poor outcomes is a hallmark of aging populations due to age-related changes of the immune response. The main driver of the physiological changes that comprise the aging phenomenon throughout the human body is the process of senescence of individual cells (1). Senescent cells are in a state of stable cell arrest triggered by a variety of mechanisms including DNA damage due to replication shortening of telomeres, stress induced senescence mediated via reactive oxygen species or oncogene induced senescence (2). Whilst incapable of replication, these cells are still metabolically active and can therefore induce changes in both the local microenvironment and systemically via secretion of cytokines and chemokines. Specifically, immunosenescence is the term that refers to the changes in immune cell function and subset composition including decreased responsiveness of B cells to stimulation; and increased activity of dendritic cells in the absence of infection leading to increased autoimmune responses (1). It is becoming increasingly clear that another important modulator of the immune response and immune cells throughout our lifespan is the human virome, which comprises a range of viruses and bacteriophage that co-exist with their host (3). Herpesviruses comprise part of this human virome and are characterized by their persistence due to their ability to establish lifelong persistent infections and thus the potential to have long term impacts on the immune system (4). Of particular interest in understanding how herpes viruses can manipulate the immune response through a lifetime of carriage is the beta herpes virus human cytomegalovirus (HCMV) – a large DNA virus that devotes a prodigious amount of genetic resources for immune modulation (5).

Primary infection with HCMV does not usually cause obvious disease in healthy people due to the induction of a comprehensive immune response involving both secreted and cellular components which controls the infection (6). However, HCMV infection can be a significant burden in immunocompromised transplant patients (7) and also causes disease when the immune system is immature such as the unborn fetus *in utero* (5). Despite this vigorous immune response in the healthy, the virus is not cleared from the host and persists as a latent infection in cells of the bone marrow and myeloid cells (8). The ability of HCMV to persist as a lifelong latent infection is likely due to the large number of immune evasion molecules encoded by the virus during both lytic and latent phases of its lifecycle (9–11). A latent infection was characterized by the presence of viral genomes in the absence of production of infectious virus but retains the capacity to reactivate (12). Whilst this remains true, it is clear that there is gene transcription (13–16) of various latency associated transcripts which act to maintain the latent phase of infection and prime the cellular environment for reactivation (17–19). Persistence of HCMV in the host is likely maintained by periodic phases of reactivation (20, 21) which are usually subclinical and controlled by the memory immune responses in the healthy (22). HCMV has been shown to perturb the composition of the memory T cell compartment in numerous independent studies [detailed here (22, 23)] resulting in expansions of CD4+ and CD8+ polyfunctional (capable of secreting different cytokines and cytotoxic functions) HCMV specific T cells with effector memory and differentiated phenotypes (24–26). These observations have been confirmed with the results from twin studies with discordant HCMV infection status between twins showing that there are increases in effector memory T cell populations and secreted cytokines in the HCMV positive twin (27).

A number of studies have implicated long-term HCMV carriage in older people with detrimental changes to the immune response, often termed the immune risk phenotype (IRP), resulting in an increased risk of all-cause mortality in over 70 year olds compared to HCMV negative individuals (28–33). The increased risk in older donors from novel infections can be exacerbated by HCMV infection, most recently seen in the COVID-19 pandemic where HCMV seropositivity has been associated with increased risk of hospitalization (34). An increased risk of mortality from cardiovascular disease has been associated with HCMV seropositivity in a number of population cohort studies in both the USA and UK (35–40) although two recently published studies did not find an association between HCMV infection and cardiovascular mortality (41, 42). The link between HCMV infection and cardiovascular disease is further supported by the association between expansions of T cell differentiated CD28 null populations in HCMV positive donors with vascular damage (43); and T cells expressing the fractalkine receptor CX3CR1 are enriched among HCMV specific T cells and can home to vascular endothelium (44). In patients who are HCMV positive there is evidence of adverse left ventricular modelling post myocardial infarction (MI) (45) and HCMV carriage increases the levels of inflammatory cytokines in chronic heart failure patients (46). HCMV DNA has also been detected in the urine (47) and blood (48) of elderly people suggesting that there is reduced control of reactivating virus, possibly due to reduced functionality of the adaptive immune response in the old. Despite this evidence, no overt disease driven directly by HCMV infection is recognized in older people, suggesting that the HCMV specific immune response retains sufficient functionality to prevent serious morbidity.

There have been a number of contradictory studies investigating whether there are expansions of HCMV specific T cells in older donors [reviewed (23)] that could contribute to the adverse impacts observed in older HCMV positive donors. It is likely that these differences are due to differences in the geographical location of the study and the socio-economic groups included. The etiology of acquisition of HCMV infection differs between high income countries, where prevalence is lower and increases with age, and low and middle income countries where there is higher seroprevalence and acquisition of the virus commonly occurs during early childhood (49). To address this issue directly we have previously investigated whether the immunomodulatory environment (10) induced by lifelong latent carriage of HCMV alters the composition and specificity of the T cell response and whether there is an effect on the carriage of latent viral genomes in the older donors. A large study cohort were recruited, encompassing young (early twenties) through to older aged donors (late seventies). Whilst we observed an expected decline in absolute naïve T cell numbers, there were no changes in magnitude of the HCMV specific T cell response with age. There were also no increases in latent viral carriage in monocytes with age, but latent virus load was positively correlated with increased size of the HCMV specific T cell response (50). This study used overlapping peptide pools of HCMV proteins as a surrogate to look at the T cell response and we had evidence that there may be a loss of functional control of viral infection *in vitro* in older donors (51).

To address the question of whether lifelong carriage of HCMV results in less functional immune responses which are less capable of controlling viral replication post reactivation in older donors, we conducted a new study: “Assessing Quality of Antiviral Responses in Ageing – AQUARIA”. Twenty-six young and old donors were recruited, and a skin punch biopsy was performed allowing the establishment of dermal fibroblast lines to use in an *in vitro* anti-viral assay we have developed – the viral dissemination assay (VDA) (51–54). We also collected blood, urine and saliva samples in order to interrogate whether there is an associated increase in *in vivo* detection of CMV DNA in the older donor cohort. Several other parameters were also measured in the AQUARIA study group, including enumeration of immune cell subset numbers in whole blood, the number of T cells producing IFNγ, TNFα and IL-10 in response to HCMV protein stimulation and the amount of HCMV specific immunoglobulins (IgG). While we did not observe significant effects of age on the magnitude of the HCMV specific T cell and total IgG responses, there was however a clear defect in the ability of the T cells from older donors to control HCMV infection and dissemination in a VDA. We also examined the ability of serum antibodies to neutralize HCMV infection of endothelial cells. This showed that neutralization capacity in older donors was significantly less effective compared to the young cohort. Finally, we looked for CMV DNA in blood, urine and saliva samples from each of the donors; we did not detect HCMV DNA in any of the young donor cohort but did detect HCMV genomes in the saliva specimens of two older donors. Together the results from the anti-viral assays and the detection of HCMV DNA *in vivo* suggests that there is a loss of T cell control of HCMV in older donors that at least in some donors can result in viral replication at peripheral tissue sites. The resulting increased viral activity and inflammation may contribute to the role of HCMV infection as a co-morbidity factor in the unwell aged.

## 2 Materials and methods

### 2.1 Ethics and donor cohort information

The donor cohort were recruited for the Assessing Quality of Anti-viral Responses in Ageing (AQUARIA) study by the National Institute of Health Research (NIHR) Cambridge Bioresource Centre (CBR) with ethical approval from the North of Scotland Research Ethics Committee 1 (NS/17/0110). Known HCMV seropositive and seronegative donors were recruited in two age groups: Young (18 – 40 years) and Old (65 years and older) and informed written consent was obtained from all participants in accordance with the Declaration of Helsinki. Volunteers were excluded from the study if they were being treated with oral or intravenous immunomodulatory drugs (including steroids, tacrolimus, cyclosporins, azathioprines, mycophenolate, methotrexate, rituximab and cyclophosphamide) within the last 3 months, undergoing injected anti-TNF treatments for rheumatoid arthritis as well as anyone receiving current or recent (last 24 months) cancer chemotherapy. Twenty-six donors had been recruited to this study by March 2020 when recruited was paused due to the COVID-19 pandemic and the introduction of government restrictions in the United Kingdom. All participants provided a 2-mm skin punch biopsy, 2ml saliva sample [collected using the Salivette^®^ system with untreated cotton swab (Sarstedt AG & Co. KG, Germany)], 5ml Urine sample and a 50ml peripheral blood sample comprising 1.2ml clotted blood, 1.2ml EDTA treated blood and 47.6ml lithium heparin treated blood samples. The characteristics (age, sex and serum HCMV specific IgG levels) of the 26 recruited AQUARIA donors are summarized in **Table 1**.

**Table 1:** AQUARIA cohort donor characteristics.

		YOUNG (<40 years)		OLD (>65 years)		All donors		All donors	
		HCMV+	HCMV-	HCMV+	HCMV-	HCMV+	HCMV-	YOUNG (<40 years)	OLD (>65 years)
Donors n (M/F)		8 (1/7)	5 (4/1)	9 (2/7)	4 (1/3)	17 (3/14)	9 (5/4)	13 (5/8)	13 (3/10)
Age (Mean ± S.D.)	Years	36.88 ± 3.14	39.80 ± 1.94	73.11 ± 2.23	75.75 ± 0.43	–	–	38.00 ± 3.21	73.92 ± 2.33
HCMV IgG (Geo Mean ± S.D.)	ISR	3.44 ± 1.95	0.57 ± 1.13	2.77 ± 1.88	0.97 ± 1.32	3.07 ± 1.90	0.72 ± 1.40	–	–

### 2.2 Isolation of human dermal fibroblasts

Primary human dermal fibroblasts were derived from the 2-mm skin punch biopsy for each donor, following the method described (55). Briefly, under sterile conditions the skin biopsy was cut into fine tissue sections and immobilized under a sterile glass coverslip and then cultured in high glucose Dulbecco’s Modified Eagle’s Medium (DMEM: Sigma-Aldrich, Poole, UK) supplemented with 20% Foetal Bovine Serum (FBS) Sera Plus (PAN Biotech UK Ltd, Wimborne, UK), 100 U/ml Penicillin and 100 μg/ml Streptomycin (Gibco, Thermo Fisher Scientific, Paisley, UK) at 37°C 5% CO_2_ in a humidified environment. Cells emerge from the tissue section after 5 days culture; initially epithelial cells grow from the section and then fibroblasts emerge from beneath the epithelial cell layer [pictured in (23)]. Once established the dermal fibroblasts lines were maintained in supplemented DMEM and once expanded cryopreserved in 10% Dimethyl sulfoxide (DMSO: Sigma-Aldrich) and 90% FBS (PAN Biotech UK Ltd).

### 2.3 Peripheral blood mononuclear cell (PBMC) isolation

Peripheral blood mononuclear cells were isolated from the heparinized blood samples using Lymphoprep (Axis-shield, Oslo, Norway) or Histopaque-1077 (Sigma-Aldrich) density gradient centrifugation. Isolated PBMC were cryopreserved in either a 10% DMSO 90% FBS solution or a serum-free freezing media composed of 60% IMDM (Iscove’s Modified Dulbecco’s Medium, Sigma-Aldrich), 10% DMSO and 30% Panexin serum replacement (PAN Biotech).

Frozen PBMC samples were removed from liquid nitrogen storage and rapidly warmed cells were immediately diluted in excess defrosting media [warmed DMEM (Sigma-Aldrich) or X-VIVO 15 (Lonza, Slough, UK) or TexMACS (Miltenyi Biotec, Woking, UK) supplemented with 10U/ml Benzonase (Merck Millipore, Dorset, UK)]. Cells were washed by centrifugation for 10 minutes at 300xg before being resuspended in X-VIVO 15 supplemented with 10U/ml Benzonase and incubated for 1 hour at 37°C. Cells were again washed by centrifugation for 10 minutes at 300xg and resuspended in TexMACS and rested overnight at 37°C prior to use.

### 2.4 Absolute count enumeration of lymphocyte subsets

The absolute number of immune cells present in whole blood samples was enumerated using Becton Dickinson Trucount tubes (BD Biosciences, Oxford, UK) following the manufacturer’s instructions. Briefly, 50µl of the EDTA treated whole blood samples was stained in the Trucount tube with a pre-mixed antibody cocktail (detailed in **Table S1**) allowing the identification of monocytes, B cells, CD4+ and CD8+ T cells and T cell memory subsets, NK cells and NKG2C+ NK cells. Following staining, the red blood cells were lysed and the cells fixed using FACS Lysing solution (BD Biosciences) before being stored at -80°C until acquisition (56). Samples were acquired on a 5-laser LSR Fortessa (BD Biosciences) with Fluorescence Minus One Controls and single color compensation controls (AbC Total Antibody Compensation Bead Kit – Thermo Fisher Scientific) utilized. Samples were analyzed and enumerated using Flowjo software v 10.8 (BD Biosciences) following the gating strategy and the formula illustrated in **Figure S1**. Results were expressed as the number of each immune cell subset per microliter of blood (cells/µl).

### 2.5 HCMV ORF peptide mixes

Libraries consisting of 15mer peptides overlapping by 10 amino acids were synthesized from 11 HCMV ORF encoded proteins [UL138, LUNA (UL81-82as), US28, UL111A (vIL-10), UL83 (pp65), UL144 (with known strain variants included), UL123 (IE1), UL122 (IE2), US3, UL82 (pp71) and UL55 (gB)] by either ProImmune PEPScreen (Oxford, UK) or JPT Peptide Technologies GmbH (Berlin, Germany) as detailed previously (50). The individual lyophilized peptides were reconstituted as described (52) and the individual HCMV ORF encoded proteins were combined into peptide pool groups consisting of 5µg/peptide/ml of (i) Latency Associated Proteins (LAT: UL138, US28, LUNA, vIL-10), (ii) pp65 and UL144, (iii) IE1 and IE2, (iv) pp71 and US3 and (v) gB proteins – the maximum number of peptides included in each pool did not exceed 200.

### 2.6 Triple Fluorospot assay

Triple Fluorospot plate kits (Mabtech AB, Nacka Strand, Sweden) were coated with capture antibodies to Human IFNγ, IL-10 and TNFα following the manufacturer’s instructions and incubated at 4°C overnight prior to use. Defrosted and rested PBMC were depleted of CD4+ T cells or CD8+ T cells by MACS using anti-CD4+ direct beads or anti-CD8+ direct beads using an AutoMACS Pro (Miltenyi Biotec) according to the manufacturer’s instructions. Efficiency of depletion and enumeration of CD3+ T cells present in each sample was determined by staining cells with a CD3-FITC, CD4-PE and CD8-PerCPCy5.5 antibody mix (BioLegend, San Diego, CA, USA) and LIVE/DEAD Fixable Far Red Dead Cell Stain (Thermo Fisher Scientific) and a known volume was acquired and analyzed by a BD Accuri C6 plus flow cytometer. A maximum of 1.5 x 10^5^ PBMC depleted of CD4+ or CD8+ T cells suspended in TexMACS were incubated in the coated FluoroSpot plates in triplicate with the five HCMV ORF peptide mixes described in section 2.5 (final peptide concentration 2 μg/ml/peptide), an unstimulated and positive control mix [containing anti-CD3 (Mabtech AB), Staphylococcus Enterotoxin B, Phytohemagglutinin, Pokeweed Mitogen, and Lipopolysaccharide (all Sigma-Aldrich)] at 37°C in a humidified CO_2_ atmosphere for 48 h. The cells and medium were decanted from the plate and the assay developed following the manufacturer’s instructions. Developed plates were read using an AID iSpot reader (Oxford Biosystems, Oxford, UK) and counted using AID EliSpot v7 software (Autoimmun Diagnostika GmbH, Straberg, Germany) using distinct counting protocols for IFNγ, IL-10 and TNFα secretion. Donor results were quality controlled as previously described (50), presented data is corrected for background cytokine secretion and expressed as spot forming units per million T cells (sfu/CD3 10^6). Previous comparison of the distribution of the response from HCMV seropositive and seronegative donors to HCMV proteins and the positive control (50) was utilized to determine the threshold of the positive response of 100 sfu/CD3+ T cells million for all 3 cytokine responses.

### 2.7 Generation of immune cell subsets

Defrosted and rested PBMC from each donor were enriched for CD4+ T cells, CD8+ T cells and NK cell subsets by MACS using CD4+ direct beads, CD8+ T cell isolation and NK cell isolation kits (Miltenyi Biotec). Cells were separated using an AutoMACS Pro Separator (Miltenyi Biotec). Efficiency of isolation of the three subsets in each sample was determined by staining cells with a CD3-FITC, CD4-PE or CD56-PE and CD8-PerCPCy5.5 antibody mix (BioLegend, San Diego, CA, USA) and LIVE/DEAD Fixable Far Red Dead Cell Stain (Thermo Fisher Scientific) and analyzed by a BD Accuri C6 plus flowcytometer. Typically, we saw 0 – 0.2% residual CD8+ T cell content in the CD4+ T cell fraction, 0 – 0.6% residual CD4+ T cells in the CD8+ T cell isolation and the mean residual CD3+ T cell content was 2.71% in the NK cell isolation.

### 2.8 Autologous viral dissemination assay

Fluorescently labelled Merlin mCherry-P2A-UL36 GFP-UL32 strain of HCMV was a kind gift from Richard Stanton, Cardiff University, UK. The generation, propagation and growth kinetics of this virus have been fully described previously (53). Human primary dermal fibroblasts (HDFs) from the AQUARIA cohort were defrosted from liquid nitrogen storage and maintained in supplemented DMEM with 20% FBS. Once revived the HDFs were seeded into half-area 96-well plates (Greiner Bio-One, Stroudwater, UK) at a density of 1 x 10^4^ cells per well. Following overnight culture in supplemented DMEM to allow the HDFs to reach confluency in the well, the cells were infected with the mCherry-GFP Merlin virus at a pre-determined low MOI (Multiplicity of Infection – typically 0.01). Twenty-four hours post infection the different immune cell subsets: total PBMC, CD4+ T cells, CD8+ T cells and NK cells were added at a range of effector to target ratios in TexMACS media and co-cultured at 37°C. After 11 days, PBMC or lymphocyte subsets were washed off and HDFs were harvested with trypsin and fixed in a 2% PFA solution for flow cytometry quantification of viral spread (**Figure S7A**). Flow cytometry analysis of the viral dissemination assay was performed on the Thermo Fisher Attune NxT flow cytometer or the BD Fortessa HTS both equipped with a blue and yellow-green laser for analyzing the GFP and mCherry signals. Data were then analyzed with FlowJo v10.8 and viral spread in each well determined as percentage of control infected wells without effector cells and control uninfected wells to determine background as previously described (53).

### 2.9 Fibroblast inhibitory molecule analysis

Dermal fibroblasts at a low passage were harvested using Accutase™ following the manufacturer instructions and washed in FACS wash Buffer (composition: 1x PBS without calcium and magnesium, 0.5% BSA, 2mM EDTA and 2mM Sodium Azide). Cells were then blocked with TruStain FcX (BioLegend) and subsequently stained with 2 panels of antibodies and matching isotype controls (**Table S3**) with brilliant stain buffer (BD Biosciences) and LIVE/DEAD™ Fixable Aqua – Dead cell stain in polypropylene tubes at 4°C. Cells were washed in FACS wash Buffer and fixed using FluoroFix Buffer (BioLegend). Samples were acquired on a 5-laser LSR Fortessa (BD Biosciences) with single color compensation controls (AbC™ Total Antibody Compensation Bead Kit and ArC™ Amine Reactive Compensation Bead kit (Thermo Fisher Scientific) and MACS Comp Bead Kit – anti-REA (Miltenyi Biotec) utilized. Samples were analyzed using Flowjo software v10.8 (BD Biosciences) following the gating strategy illustrated in **Figure S2**, the geomean fluorescence intensity was normalized to the corresponding isotype and normalized expression over 100 units was deemed positive expression of each marker as described (57).

### 2.10 HCMV IgG antibody levels protocols

Human cytomegalovirus serostatus was confirmed and quantified using serum from the clotted blood sample and HCMV IgG levels determined using an IgG enzyme-linked immunosorbent (EIA) assay, HCMV Captia (Trinity Biotech, County Wicklow, Ireland), following the manufacturer’s instructions. The EIA assay is semi-quantitative, containing negative, positive and calibrator controls allowing the computation of an immune status ratio (ISR) value for the amount of anti-HCMV IgG present in the sample, CMV negative serostatus was determined with an ISR value of less than 0.9 and positive serostatus with an ISR greater than 1.1.

Total IgG antibodies for HCMV gB and pentamer protein were measured using an adapted previously described method (58, 59). Briefly, a high binding ELISA 96-well plate was coated with 0.75µg/ml HCMV gB (ab43040) (Abcam, Cambridge, MA) or Pentamer protein (The Native Antigen Company, Upper Heyford, UK) in coating buffer (pH9.4-9.8) and incubated overnight at 4°C. Plates were blocked with 2% fetal calf serum in PBS for 1 hour at 37°C then washed with PBS supplemented with 0.1% tween-20. Serum dilutions in blocking buffer were then added to the wells and incubated for a further hour at 37°C. Unbound antibody was removed by washing and peroxidase-conjugated secondary antibody (goat-anti-human IgG, Dianova) was added for 1 h, 37°C. After washing, 100 µl of tetramethylbenzidine peroxidase substrate was added to each well for 30 minutes, diluted 1:1 in peroxidase substrate solution B (KPL, USA). The reaction was stopped by adding 100 µl of 1M phosphoric acid to each well. The optical density at 450 nm (OD450) was determined using an Emaxmicroplate reader (Eurofins MWG Operon). Previously determined CMV negative serum was set as baseline.

### 2.11 Neutralization assays

Measurement of the functional capacity of CMV specific antibodies in AQUARIA donor serum samples was performed using neutralization assays with infected autologous human dermal fibroblasts (HDFs) or Adult Retinal Pigment Epithelial (ARPE-19) cells. 1 x 10^4^ cells/well were seeded in a half-area 96 well plate and incubated overnight. In order to assess neutralizing capacity, the dual tagged Merlin virus (for infecting the HDFs) or TB40\E-UL32-GFP (a gift from Dr Christian Sinzger, University of Ulm for infecting the ARPE-19 cells) at a MOI of 1.0 was pre-incubated with heat-inactivated sera over a range of dilutions for 1 hour at room temperature. The sera/virus cocktail was transferred onto the HDF or ARPE cells in triplicate and rocked for 3 hours at room temperature. Media was removed and the cells washed with PBS and fresh media replenished. After 72 – 96 hours culture the adherent cells were trypsinized and fixed in a 2% paraformaldehyde solution and analyzed by flow cytometry as previously described. Data were then analyzed with FlowJo v10 and the percentage viral infection in each well was normalized to the infected control wells.

### 2.12 DNA extraction from whole blood, saliva and urine samples

For each donor the provided biological samples of whole blood, saliva and urine samples were processed on the day of collection, a 1ml EDTA treated whole blood sample was stored and the urine sample was aliquoted into a maximum of 3 x 5ml cyrovials under sterile conditions. Following collection, the Salivette^®^ tube containing the saliva sample was processed by centrifugation at 1000xg for 2 minutes and the recovered saliva transferred to 2ml cyrovials in a class II MSC. All biological samples were then stored at -20°C until DNA extraction. Prior to DNA extraction 0.5ml of the saliva sample [diluted to 4ml with PBS (Thermo Fisher Scientific)] and 4ml of the urine sample for each donor were concentrated using Amicon Ultra-15 10K Centrifugal Filter Devices (Sigma-Aldrich) to a 200µl and 140µl sample size respectively. DNA was extracted from the 1ml blood using QIAamp DNA Blood Midi kit (Qiagen, Manchester, UK) following the manufacturer’s instructions. The concentrated saliva sample was processed using the QIAamp DNA Blood Mini kit (Qiagen) using the instructions in appendices F and K to maximize viral DNA recovery. DNA was extracted from the concentrated urine samples using the QIAamp Viral RNA Mini kit (Qiagen) using the protocol “Purification of Cellular, Bacterial or Viral DNA from Urine” as the buffer used in this procedure inactivates PCR inhibitors known to be found in urine.

### 2.13 Measurement of HCMV DNA in biological specimens

Real time quantitative PCR was performed on the extracted DNA samples using a StepOne Plus Real Time PCR system (Applied Biosystems, Thermo Fisher Scientific) using a method adapted from (60). Amplification of HCMV DNA used glycoprotein B primers (61) and detection with a Taqman probe (60) (specific sequences are detailed in **Table S2**) mixed with ABI Universal Mastermix (Applied Biosystems, Thermo Fisher Scientific). The final assay volume comprised 25µl with a 5µl extracted DNA sample or control sample included. PCR cycling conditions were 2 minutes at 50°C, 10 minutes at 95°C and 45 cycles of 15 seconds at 95°C and 60 seconds at 60°C. All donor samples were screened in triplicate with a standard curve of 1 – 10^4^ HCMV genomes [WHO International Standard (62)] and spiked positive and negative HCMV DNA controls from blood, saliva and urine were generated as previously described (50) and run alongside the unknown samples. HCMV DNA copies detected was calculated using the StepOne software (Applied Biosystems, Thermo Fisher Scientific) from the standard curve run cycle threshold (Ct) values and required a minimum of 2/3 positive wells to report a result expressed as HCMV copies per milliliter.

### 2.14 Statistics

Statistical analysis was performed using GraphPad Prism version 8 and 9 for Windows (GraphPad Software, San Diego, CA, USA). Absolute count data was transformed and analyzed by ordinary 1-way ANOVA with *post hoc* Fisher’s LSD test to compare groups. Fluorospot data was transformed and analyzed by ordinary 1-way ANOVA with *post hoc* Bonferroni’s multiple comparisons test. The normalized viral spread data was plotted for each immune cell subset and the area under the curve (AUC) calculated (illustrated in **Figure S7C**). The calculated AUC data was compared between groups by 2-way ANOVA with multiple comparisons controlled by the False Discovery Rate using the two stage step up Method of Benjamini, Krieger and Yekuteli and geomeans and 95% Confidence intervals of each group calculated. The comparison of the expression of inhibitory molecules on dermal fibroblasts derived from young and old donors was performed by multiple unpaired t-test of the transformed normalized fluorescence. The serum dilution curves from the gB and pentameric IgG ELISAs had AUC calculated; absolute IC50 curves were fitted where possible to the triplicate neutralization data and AUC calculated. The antibody quantification and neutralization data were compared using a one-way ANOVA Kruskal–Wallis test with multiple comparisons controlled by the False Discovery Rate. A non-linear Sigmoidal 4-parameter logistic curve was fitted to the qPCR standard curve.

## 3 Results

### 3.1 Characterization of the AQUARIA study cohort

We have previously demonstrated that neither the magnitude nor breadth of HCMV specific T cell responses (following stimulation with overlapping peptide pools specific for multiple HCMV proteins) were diminished with increasing donor age (50). Whilst this approach can enumerate these responses and determine secretion of antiviral effectors such as IFNγ, TNFα and immune suppressive IL-10 it does not assess antiviral effector function in the context of HCMV infected cells where the virus is expressing the full range of its immune evasion genes. The aim of this study was to investigate whether the functionality of the HCMV specific immune response was affected by donor age using an *in vitro* viral dissemination model to interrogate the ability of different immune cell populations to control viral infection in combination with quantifying HCMV DNAemia and viral loads in peripheral sites (saliva and urine). A cohort of 26 donors was recruited according to the criteria detailed in the methods and provided a 2mm skin punch biopsy enabling the establishment of autologous dermal fibroblast lines from each donor. The age, sex and total HCMV IgG levels for the AQUARIA study cohort are summarized in **Table 1**; in this study all the old donors were aged over 70 years at the time of recruitment.

A detailed phenotypic analysis was performed on whole blood from all donors enabling the determination of absolute counts of different cellular subsets. Total CD4+ and CD8+ T cells in addition to memory and differentiation subsets, monocytes, B cells and NK cells were enumerated; the data is summarized in **Figures S3–S5**. The results show that numbers of CD14+ monocytes were significantly increased (**Figure S3A**) and B cells (CD19+ CD3- cells) (**Figure S3B**) were significantly decreased in HCMV seropositive donors irrespective of donor age. NK cell numbers were not significantly different between HCMV positive and negative donors but there was a trend towards lower numbers of NK cells in HCMV seropositive donors (**Figure S3E**). Therefore, when comparing the proportion of NKG2C+ NK cells between HCMV seronegative and seropositive donors there was a significant increase in this population in the HCMV seropositive donors (**Figure S3H**), a phenotype previously described in many different donor cohorts (63, 64).

Examination of CD4+ T cell numbers revealed that there was a significant decrease in total CD4+ T cells in the HCMV seropositive group irrespective of age (**Figure S4A**) and also a significant decrease in activated CD4+ T cells (HLA DR+) in the same group (**Figure S4B**). Analysis of the memory subsets defined by expression of CD27 and CD45RA (T_NAIVE_: CD45RA+ CD27+; TCM: CD45RA- CD27+; TEM: CD45RA- CD27-; TEMRA: CD45RA+ CD27-) showed a significant decrease in naïve T cell numbers in old CMV seropositive donors (**Figure S4D**). Highly differentiated T cells can be defined by the loss of expression of CD28 and gaining expression of CD57, in the CD4+ T cells this subset was significantly increased in all the HCMV seropositive donors irrespective of donor age (**Figure S4G**). There were no differences in the numbers of CD8+ T cells between the HCMV serostatus age groups (**Figure S5A**), there was however a significant decrease in naïve CD8+ T cells with donor age (Young CMV+ vs Old CMV+) as well as a significant decrease in naïve CD8+ T cell numbers in all HCMV positive donors compared to HCMV negative (**Figure S5D**). These results show that there is a decrease in the naïve T cell pool and an increase in differentiated T cells that is a well-established phenotype in long-term carriage of HCMV (65).

### 3.2 T cell cytokine responses to HCMV peptide pool stimulation

**Figure 1:**
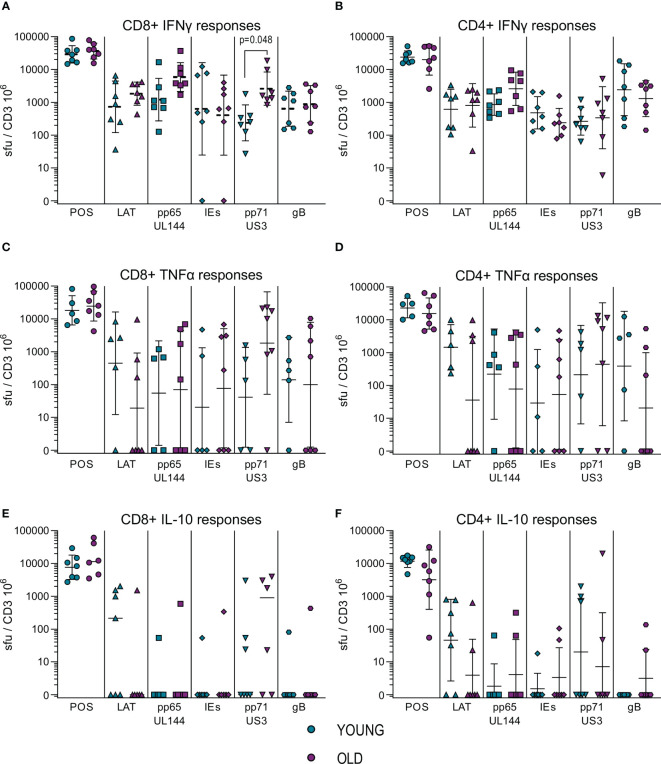
Magnitude of T cell IFNγ, TNFα and IL-10 responses to HCMV protein stimulation. The cytokine secreting CD8+ and CD4+ T cell response to five HCMV protein mixes and positive control stimulation were measured in the 17 HCMV seropositive donors by triple color fluorospot. The fluorospot results have been converted into spot forming units per million CD3+ T cells (sfu/CD3 10^6^) with background counts for each cytokine subtracted. The cytokine response to the positive control, latency associated proteins (LAT: UL138, US28, LUNA, vIL-10), pp65 and UL144, IE1 and IE2 (IEs), pp71 and US3 and gB proteins are shown for both young (turquoise points) and old (purple points) donors for CD8+ T cells (PBMC with CD4+ T cells depleted) and CD4+ T cells (PBMC with CD8+ T cells depleted. The IFNγ CD8+ **(A)** and CD4+ T cell **(B)** responses, TNFα CD8+ **(C)** and CD4+ T cell **(D)** responses and IL-10 CD8+ **(E)** and CD4+ T cell **(F)** responses are shown (with the geomean and geometric standard deviation (S.D.) indicated for each group). The fluorospot cytokine data was transformed and analyzed by ordinary 1-way ANOVA with post-hoc Bonferroni’s multiple comparison tests to compare young and old responses to each protein mix stimulation for each T cell subset and cytokine, significant differences are marked on the graph with the p-value indicated.

To further characterize our immune cell populations, we measured the frequency HCMV specific IFNγ, IL-10 and TNFα producing CD4+ and CD8+ T cells using a combination of five HCMV protein peptide pools, representing the latency associated transcript (LAT) proteins (UL138, LUNA, US28 and vIL-10), pp65 and UL144 proteins, IE1 and IE2 (IEs) proteins, pp71 and US3 proteins and gB protein. **Figure 1** summarizes the spot forming units (sfu) per million CD3+ T cells responses generated by the HCMV seropositive donors split into young and old donors for both CD4+ (right-hand column) and CD8+ (left-hand column) T cells for all 3 cytokines. The results show that there were strong IFNγ responses (**Figures 1A, B**) to all HCMV proteins stimulation from both old and young donors; all of the cohorts producing above threshold responses to at least 3 out of 5 of the HCMV protein pools (**Figure S6A**). There were no differences between the young and old cohort in the amount of IFNγ produced by CD4+ T cells in responses to HCMV protein stimulation, however in the CD8+ T cell subset there was a significant increase in the production of IFNγ by the older donors in response to pp71 and US3 stimulation. All of the five HCMV protein mixes were capable of generating a TNFα T cell response in at least half of all donors regardless of age (**Figures S6B, C**). The vast majority (11/12 CD8+ and CD4+ T cells – **Figure S6A**) of the donors in this study produced TNFα in response to stimulation by at least one of the HCMV protein mixes. Whilst there is variation in the mean observed between the young and old groups (**Figures 1C, D**) there is no significant difference in the magnitude of the TNFα response between the young and old donors in this study cohort.

Production of IL-10 in response to HCMV protein stimulation was at a lower frequency in numbers of responding donors (**Figure S6**) compared to the IFNγ and TNFα cytokine responses. IL-10 was produced by both CD4+ and CD8+ T cells in response to stimulation with the LAT protein mix and the pp71 and US3 proteins mix (**Figures 1E, F**, **S6B, C**), with pp71 and US3 protein stimulation resulting in a considerable amount of IL-10 production from both T cell subsets (**Table S4**). There was no defect in either the magnitude or breadth of the IL-10 T cell responses to HCMV in the old as compared to the young and production of IL-10 by both CD4+ and CD8+ T cells replicates our observations in a previous cohort analysis (50, 51). Overall, the results from measuring IFNγ, TNFα and IL-10 T cell responses to stimulation with HCMV protein mixes of overlapping peptides did not show a major effect of age on magnitude, breadth or frequency of the cytokine responses. This result for IFNγ and IL-10 responses is in agreement with a previous independent cohort analysis (50, 51). Overall, the cytokine production data in response to HCMV protein overlapping peptide pools shows that both young and old CMV seropositive donors have high frequency IFNγ and TNFα anti-viral T cells and we see a similar pattern of expression of the immunosuppressive IL-10 in response to specific groups of HCMV peptides between the two age cohorts. A lack of differences in surrogate markers of T cell function led us to investigate direct effector functions of CD4+ and CD8+ T cells in response to HCMV infection.

### 3.3 The anti-viral T cell effector functionality is diminished in older donors

The use of overlapping peptide pools of viral proteins is useful in mapping out T cell responses but is limited in determining T cell functionality in response to lytic cycle infected cells where HCMV can express its full range of immune evasion genes. In order to determine and quantify the anti-viral effector function of the immune cells derived from the AQUARIA donor cohort, we have utilized an autologous *in vitro* HCMV dissemination assay that we have previously developed (51–54) termed the viral dissemination assay (VDA). Utilizing HCMV infected dermal fibroblast lines, grown from the individual donor skin biopsy, allowed investigation of the capacity of whole PBMC as well as isolated CD4 and CD8+ T cells and NK cells to control viral spread in a fully autologous setting. HCMV infections were performed with a dual tagged Merlin HCMV strain which expresses mCherry linked to UL36 (an immediate early protein) and UL32-GFP fusion (pp150 a true HCMV late gene). When fibroblasts are infected with a low MOI the virus spreads through the fibroblasts over time, this virus has been characterized and the kinetics of expression of mCherry at early time points and GFP and mCherry at late times of infection over a twelve day time-course has been measured (53). The mCherry signal is visible from 18 hours post infection by fluorescent microscope observation with increasing expression with time; by about 72 hours post infection the mCherry+ cells become GFP+ as they proceed through HCMV DNA replication and expression of late genes with the assembly and release of new virions. New virions infect surrounding uninfected cells which can be seen by the subsequent temporal expression of mCherry and then GFP. The functions of the proteins encoded by the two viral genes tagged in this strain of virus used also allow us to determine whether the virus has entered the cell and initiated viral gene expression (UL36 mCherry expression) and whether this results in viral DNA replication and production of infectious virion progeny (UL32 GFP expression along with continuing mCherry). The VDA assays presented here were co-cultured with different immune cell subsets for between 9 – 12 days, which in the virus only control wells allows enough time for significant viral replication and spread. **Figure S7A** shows the appearance of the infected control at this timepoint, where the two phases of infection are clearly observed.

**Figure 2:**
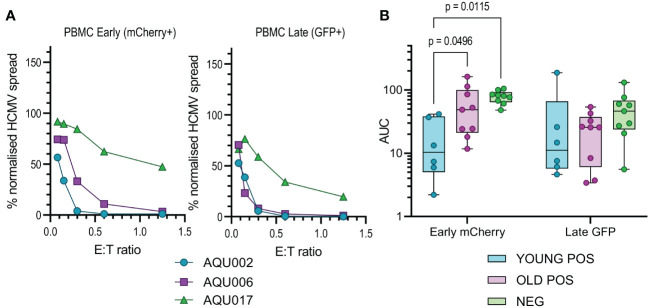
Anti-viral activity of whole PBMC from Young and Old Seropositive and Seronegative donors. Untouched donor PBMCs over a range of effector:target (E:T) ratios were co-cultured with autologous dermal fibroblasts infected with the dual fluorescence tagged Merlin strain of HCMV. After 11 days the cultures were harvested and analyzed for mCherry and GFP expression by flow cytometry. Representative curves of normalized viral dissemination for a young seropositive (AQU002), old seropositive (AQU006) and seronegative (AQU017) donors for both Early (mCherry+) and late (GFP+ and mCherry+) infection are shown **(A)**. Areas under the curve (AUC) were calculated for all donors and the results grouped according to age and serostatus and presented as a min – max box and whiskers plots with median and upper and lower quartiles indicated **(B)**. The calculated AUC data was compared between groups by 2-way ANOVA with multiple comparisons, controlled by the False Discovery Rate using the two stage step up method of Benjamini, Krieger and Yekuteli, performed to compare the three groups (Young Pos, Old Pos and Neg) at both viral infection timepoints. Significant differences between the groups are shown as the p-value.

**Figure 3:**
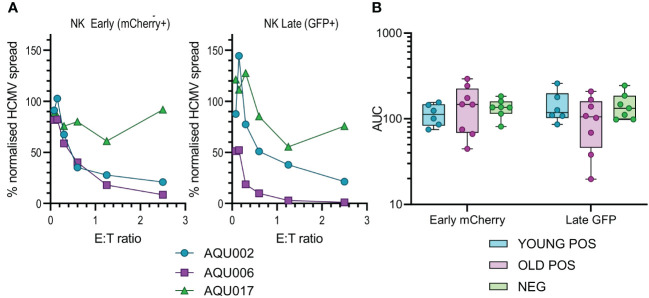
Anti-viral activity of isolated NK cells from Young and Old Seropositive and Seronegative donors. Isolated NK cells were co-cultured with infected autologous dermal fibroblasts as described in **Figure 2**. Representative curves of normalized viral dissemination from the young (AQU002), old (AQU006) and seronegative (AQU017) donors are shown for both Early and Late infection **(A)**. Calculated AUC for all donors are shown grouped according to age and serostatus and presented as a min – max box and whiskers plot with median and upper and lower quartiles indicated **(B)**. The NK cell AUC data was compared by 2-way ANOVA with multiple comparisons, there were no significant differences in the ability of NK cells to control viral dissemination between seropositive and seronegative donors.

**Figure 4:**
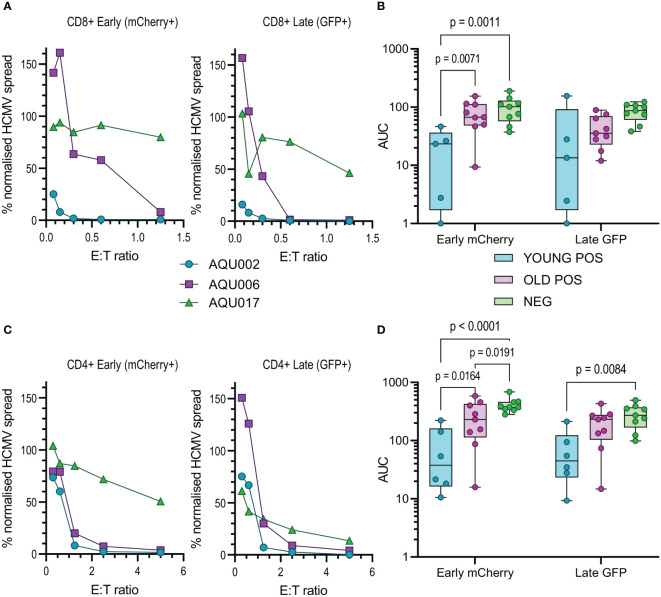
Anti-viral activity of isolated CD8+ and CD4+ T cells from Young and Old Seropositive and Seronegative donors. Isolated CD8+ T cells or isolated CD4+ T cells were co-cultured with infected autologous dermal fibroblasts as previously described. Shown are representative curves of normalized viral dissemination from the young (AQU002), old (AQU006) and seronegative (AQU017) donors for the CD8+ **(A)** and CD4+ **(C)** T cell co-culture. AUC were calculated for all donors and the results are summarized graphically for CD8+ **(B)** and CD4+ **(D)** T cells, the data is presented as a min – max box and whiskers plots with median and upper and lower quartiles indicated. 2-way ANOVA with *post-hoc* multiple comparisons controlled by the False Discovery Rate was performed to compare young and old seropositive donors and the seronegative donor results. Significant differences between the three groups are shown on the graphs as the p-value.

Autologous fibroblasts were infected and co-cultured with either total PBMC, purified NK cells, CD4+ T cells or CD8+ T cells at a range of E:T ratios; wells with no immune cells added acted as an untreated control to determine the maximum virus spread during the assay. Fibroblasts were harvested and analyzed for the expression of mCherry and GFP by flow cytometry and the data was normalized to the untreated controls. The percentage normalized HCMV infected cells in the early (mCherry+ GFP-) and late (mCherry+ GFP+) phases of infection were plotted against the effector to target (E:T) ratios (PBMC co-culture example of early phase infection: **Figure S7B**). The average response of the four different cellular subsets, PBMC, NK cells, CD8+ and CD4+ T cells from the three different cohorts over the range of E:T ratios examined are illustrated in **Figure S8** and representative young and old seropositive and seronegative donor responses are shown in **Figures 2A**, **3A** and **4A, C**. To enable statistical comparison of the ability of different donors immune cells to control viral dissemination over the same range of E:T ratios the area under the curve (AUC) was calculated (shaded area in **Figure S7C**). The AUC value enables direct comparison of the four different immune cell populations derived from young and old HCMV seropositive donors and HCMV seronegative donors to control viral dissemination. A lower AUC value indicates more effective control of *in vitro* viral infection.

The calculated AUC values from the early and late gene phases were collated for each of the four cellular subsets examined. The collated results are shown in box and whisker plots for total PBMC (**Figure 2B**), NK cells (**Figure 3B**), CD8+ (**Figure 4B**) and CD4+ T cells (**Figure 4D**) from HCMV seronegative, young HCMV seropositive and old HCMV seropositive donors. The results show that that total PBMC derived from young HCMV seropositive donors are significantly better at controlling viral spread (mCherry+ GFP- cells) than the old seropositive donors and seronegative donors. There was no significant difference in the inhibition of late gene expression although the trend was that PBMC derived from HCMV positive old or young donors were more suppressive than those derived from HCMV seronegative donors. PBMC is composed of CD4+ and CD8+ T cells as well as innate immune effectors such as NK cells and monocytes. Donors that are HCMV seronegative will not have developed HCMV specific memory T cell responses, explaining the poor ability of PBMC from these donors to control viral spread. However, the inability of PBMC from older HCMV positive donors to control mCherry expression compared to younger HCMV positive donors was striking and pointed towards a possible defect in HCMV specific memory T cell response.

The results from the purified NK cell co-cultures demonstrate that there is no difference in the level of control of viral spread or suppression of late gene expression between NK cells from CMV seropositive or CMV seronegative donors. It was noted that there is a range of NK cell effector function between the different donors but this range is overlapping in all three groups. Given the previous results with total PBMC, this suggests that there may be defects in the T cell effector function in the elderly cohort. This conclusion was verified when looking at the results from the co-culture of purified CD8+ and CD4+ T cell subsets with autologous HCMV infected fibroblasts. Analysis of this data clearly demonstrates that both T cell subsets derived from the young seropositive donors were significantly better at controlling viral dissemination than the old donors (**Figures 4B, D**). The ability to suppress late gene expression is not statistically significantly better for the CD8+ T cells but is clearly demonstrated by the CD4+ T cells from young donors. Taken together the analysis of NK and T cells indicate that the adaptive immune response in older donors is less effective in controlling HCMV dissemination. Correlation of the magnitude of the total HCMV specific CD8+ and CD4+ T cell response (illustrated in **Figure 1**) with the anti-viral activity of the T cell subsets did not reveal a significant association between an expanded HCMV specific T cell population and effective control of viral spread (**Figure S9**).

**Figure 5:**
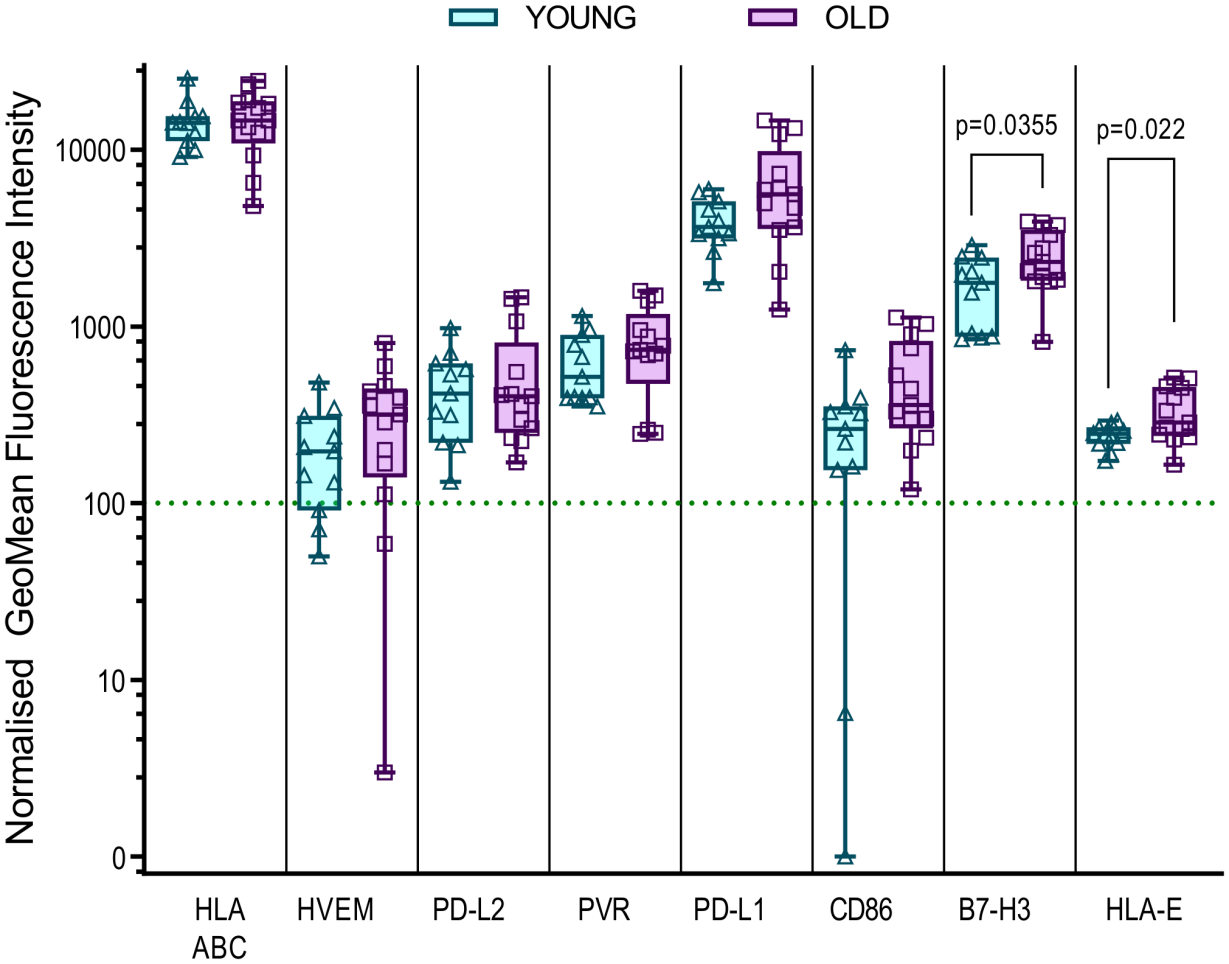
Expression of inhibitory ligands by Young and Old AQUARIA human dermal fibroblasts. The AQUARIA cohort derived dermal fibroblasts were analyzed for the expression of inhibitory ligands by flow cytometry. Geomean fluorescence intensity (gMFI) was normalized to the corresponding isotype and normalized expression greater than 100 units was deemed as positive cell surface expression of the protein. Summarized is the normalized gMFI expression of MHC Class I (HLA-ABC), HVEM (CD270), PD-L2 (CD273), PVR (CD155), PD-L1 (CD274), CD86 (B7-2), B7-H3 (CD276) and HLA-E for each age group presented as box and whisker min – max plots with median and quartiles indicated. Multiple unpaired t-tests of the transformed normalized expression data were performed and the significant differences between the age cohorts are indicated as the p-value. There is no significant difference in the expression of MHC Class I between the young (Turquoise symbols) and old (purple symbols) fibroblasts. The remaining molecules are arranged from left to right according to the increased expression of the molecule by the older donor dermal fibroblasts, with significant differences observed between the expression of B7-H3 and HLA-E.

It is clear that the age associated decline in the immune responses encompasses many factors, there is evidence from other studies that defects in the local microenvironment, such as the skin, may affect the outcomes of an immune response. An increase in senescent fibroblasts in the skin results in the recruitment of monocytes which inhibit the antigen specific response (66) and increased expression of HLA-E, which can interact with the inhibitory molecule NKG2A, is a marker of these senescent fibroblasts in older humans (67). We therefore hypothesized that the differences in immune control observed could potentially be linked to the possibility that dermal fibroblasts derived from the older donors in this study may have increased expression of inhibitory molecules that affect the ability of older HCMV positive donors to control viral spread. To investigate this, we measured the expression levels of fifteen different inhibitory ligands molecules alongside fibroblast lineage markers to determine which molecules are expressed on dermal fibroblasts and whether the expression differs between old and young donors. We identified eight molecules of the sixteen measured which were expressed by the twenty-five dermal fibroblast lines examined (**Figure S10**), MHC Class I (HLA ABC), HVEM (CD270), PVR (CD155), PD-L1 (CD274) and PD-L2 (CD273), CD86 (B7-2), B7-H3 (CD276) and HLA-E. The expression of these eight molecules on dermal fibroblasts derived from young and old donors in the AQUARIA cohort are summarized (**Figure 5**). There was no age difference in expression of MHC Class I molecules, but there was a trend towards increased expression of HVEM, PD-L2, PVR, PD-L1 and CD86 on dermal fibroblasts from old donors. The orphan ligand B7-H3 and HLA-E were significantly increased on older donor dermal fibroblasts.

### 3.4 Antibody neutralization of HCMV infection is diminished in older donors

**Figure 6:**
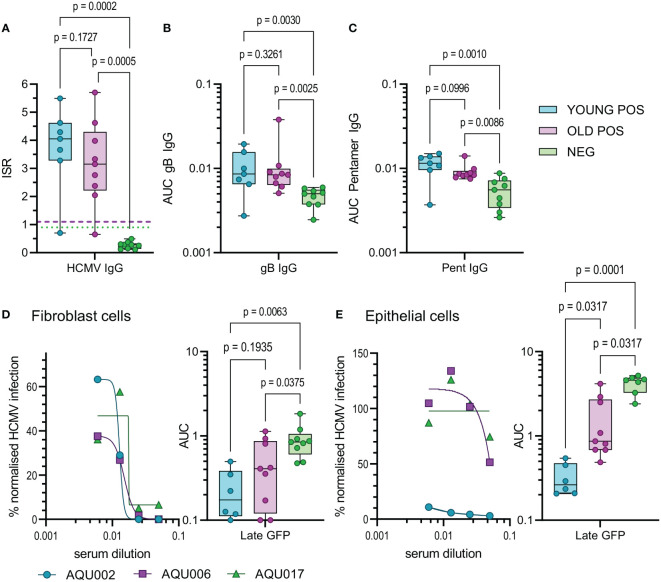
HCMV Antibody quantification and neutralization capacity of young and old seropositive and seronegative donors. The amount of antibody specific to HCMV was quantified in the sera from the AQUARIA donor cohort. The amount of IgG reacting to the virus was first quantified by the Captia HCMV EIA with results expressed as an immune status ratio (ISR) **(A)** the negative boundary of 0.9 (green dotted line) and positive boundary of 1.1 (pink dashed line) are shown for reference. The amount of IgG antibodies in donor sera reactive to gB protein **(B)** and the pentameric complex **(C)** are shown as AUC calculated from OD 450 values from a range of serum dilutions (1:100 – 1:10000). Neutralization assays with heat inactivated sera were performed on fibroblasts **(D)** and epithelial cells **(E)**, serum was diluted and pre-incubated with the fluorescence tagged virus prior to adding to the cells. Representative curves for young (AQU002), old (AQU006) seropositive and seronegative (AQU017) donors are shown for late gene GFP expression for both cell types. AUC were calculated for all donors and the results for the three age and serostatus groups are shown and presented as a min – max box and whiskers plots with median and upper and lower quartiles indicated. All data was analyzed using a one-way ANOVA Kruskall-Wallis test with *post-hoc* multiple comparisons controlled by the False Discovery Rate. The p-values for all these comparisons are shown on each graph, a significant difference is a result of p<0.05.

Finally, we examined aspects of the humoral response in young and old donors. HCMV infection induces antibody responses some of which have the capacity to neutralize HCMV infection. Using a diagnostic HCMV ELISA we determined the total HCMV specific IgG (measured as the mean of the Immune status ratio) of the donor cohort, (summarized in **Table 1** and **Figure 6A**). There was no significant difference in the amount of CMV specific IgG measured between the young and old CMV seropositive donors. Analysis of the amount of IgG specific to the gB protein and the pentameric complex [gH/gL/pUL128-130-131 (68)] of HCMV were also analyzed in this cohort. The results show that as expected young and old CMV seropositive donors have significantly more of both gB specific (**Figure 6B**) and pentamer specific (**Figure 6C**) antibodies compared to the seronegative cohort. There were however no significant differences in the amount of these antibodies between the young and old seropositive cohorts, although we noted a trend towards lower amounts of both gB and Pentamer specific IgG antibodies in the older donor cohort.

The measurement of HCMV specific IgG levels does not measure the effector function of these antibodies, an important characteristic is the ability to neutralize HCMV infection. As such, we also performed neutralization assays against HCMV infection of both fibroblasts and ARPE-19 cells in order to measure the capacity of both trimer specific and pentamer specific neutralizing antibodies (68). The percentage of infection achieved in the presence of serially diluted complement inactivated donor serum was measured and the area under the curves calculated. Antibodies from CMV positive donors were significantly better at neutralizing infection in fibroblasts compared to seronegative donors as expected (**Figure 6D**), however there was no statistical difference between the young and old seropositive cohorts. Strikingly, neutralization of the HCMV infection of ARPE-19 cells (an indication of neutralizing anti-pentamer antibodies) was clearly superior and significantly higher in serum from the young donors compared to the old donors (**Figure 6E**). The avidity of the binding of the serum IgG specific to both gB and the pentameric complex from the young and old HCMV seropositive donors were measured. By using urea treatment to displace weakly bound antibody the percentage loss of HCMV specific IgG can be quantified (**Figure S11C**). There were no differences in the percentage loss of antibodies specific to both HCMV proteins between the young and old HCMV seropositive donors. This suggests that the neutralizing defect we observed is not due to poor avidity in the old donor cohort as the pentamer specific IgG binding did not differ between the age groups and the percentage loss was increased in the young donors compared to the old.

### 3.5 *In vivo* detection of CMV genomes: CMV genomes are detected in saliva of older donors

**Figure 7:**
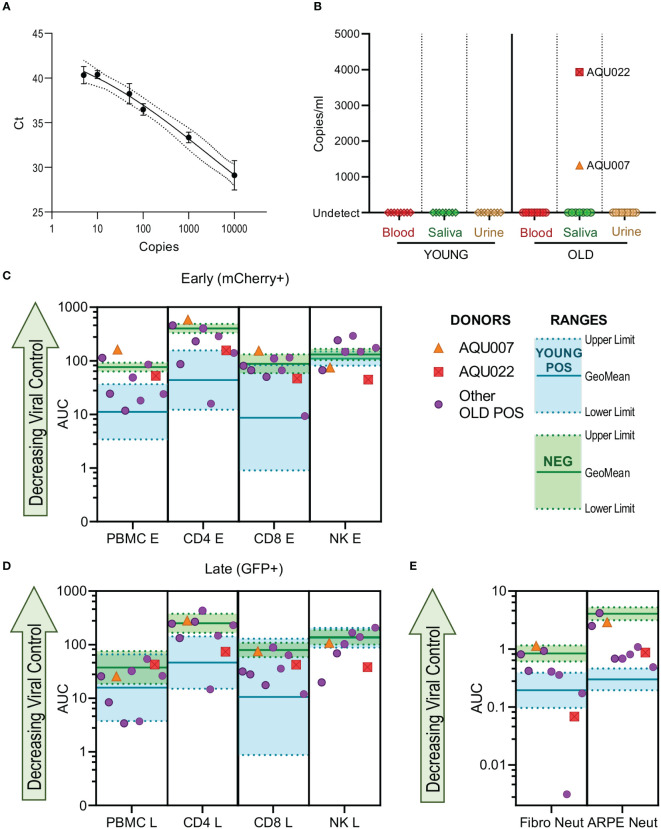
HCMV genomes are detected in the saliva of older AQUARIA donors. Realtime quantitative PCR was performed on extracted DNA samples from all donors’ biological samples. The Ct values of the standard curve of the HCMV genomes is shown **(A)** showing detection to 5 genome copies. HCMV copies per ml were calculated using the standard curve in the Step One software as described in the methods. No HCMV DNA was detected in the CMV seronegative donors summarized **(B)** are the results for the Young and Old HCMV positive donors Blood, Saliva and Urine samples. CMV genomes were detected in the saliva of 2 old donors AQU007 (orange triangle) and AQU022 (red square). The anti-viral cellular results of the AQUARIA cohort are summarized for early gene **(C)** and late gene **(D)** phases of viral infection and antibody neutralization **(E)** showing the other individual Old Positive donors (purple symbols) AUC responses and the geomean and range of the young positive (turquoise) and negative donor (green) responses illustrated, the responses of donors AQU007 (orange triangle) and AQU022 (red square) are highlighted for each cellular category and antibody neutralization. The young seropositive donor range of response generally represent anti-viral control and the negative donor range of responses reflect a loss of anti-viral control the trend of decreasing control of the virus is shown as the green arrow on each graph.

HCMV DNAemia is detected in immune suppressed transplant patients following primary infection or reactivation but also at peripheral sites e.g., salivary glands and the kidney. We hypothesized that if elderly HCMV seropositive individuals had defects in effector function of HCMV specific immune response this might be reflected *in vivo* by the detection of HCMV in blood, saliva or urine. These samples were taken from all 26 members of the cohort, following DNA extraction a quantitative real-time PCR assay detecting HCMV DNA was performed on the biological samples. The assay was able to detect a minimum of 5 genome copies (**Figure 7A**). HCMV copies per ml in blood, urine and saliva were calculated from a standard curve, no HCMV DNA was detected in the HCMV seronegative donors. No HCMV genomes were detected in any of the three biological samples in the young donors, HCMV genomes were however detected in the saliva sample from two old donors (1317 and 3930 copies per ml for donors AQU007 and AQU022 respectively) (**Figure 7B**).

We compared these HCMV saliva positive older donors anti-viral functionality and immune cell composition to the remainder of the older donor cohort and young and seronegative donors to see if this provided an explanation for the detection of virus. The viral dissemination (in co-culture assays with PBMC, NK cells, CD4+ and CD8+ T cells) and antibody neutralization results of the individual older donor cohort are shown with the range of the young donor (better viral control) and the seronegative donors (decreased viral control) indicated with the AQU007 and AQU022 responses superimposed on these charts for both phases of infection (**Figures 7C, D**). Both donors with detectable HCMV DNA *in vivo* had poor PBMC control of viral spread, overlapping the seronegative range, at both phases of infection. AQU007 CD4+ and CD8+ T cell control of viral spread was poor and worse than the seronegative donor cohort at the early phase of infection and overlapped with the seronegative range at late phases of infection, whereas AQU022 AUC values for both T cell subsets overlapped with the young CMV positive range. Both donors had better NK cell control of viral dissemination than most of the other older donors and the young donor cohort at both phases of infection (**Figures 7C, D**). When analyzing the antibody neutralization results AQU007 had poor neutralization of HCMV infection of both fibroblasts and ARPE-19 cells, however, AQU022 demonstrated extremely good neutralization of infection in fibroblasts with poorer pentamer neutralizing antibodies than the young range (**Figure 7E**). Analysis of the absolute numbers of the major immune cell subsets showed that donors AQU007 and AQU022 have low numbers of B cells and T cells compared to the other older CMV positive donors (**Figure S12**).

## 4 Discussion

The aim of this study was to investigate if there is an effect of donor age on the control of HCMV by examining peripheral sites for evidence of HCMV replication *in vivo* in addition to measuring the anti-HCMV cellular and humoral immune responses *ex vivo*. HCMV seropositive and seronegative healthy donors were recruited in two age cohorts of young (aged 40 years and younger) and old (aged 65 years and older) donors allowing the effect of age on the measured parameters to be performed. Recruitment to the study was paused in March 2020 due to United Kingdom government national lockdown measures in response to the COVID-19 pandemic, therefore all the samples analyzed in this study were likely unaffected by infection with SARS-CoV2.

Using a comprehensive antibody phenotyping panel, we enumerated the absolute numbers of CD14+ monocytes, CD19+ B cells, CD56 and CD16 defined NK cells and total CD3+ T cells in addition to the CD8+ and CD4+ memory and differentiation defined T cell subsets. This revealed that within the AQUARIA cohort there was a significant impact of age on the numbers of naïve CD8+ T cells present in the peripheral blood, which is consistent with observations we and others have previously seen that have shown a loss of naïve T cell numbers with increasing age (50, 69–71). HCMV seropositivity irrespective of age made a significant difference to the size of various different T cell populations including significantly decreasing the numbers of naïve CD4+ and CD8+ T cells and increasing the numbers of highly differentiated (measured by loss of CD28 expression) T cells. An increase in the proportion and numbers of differentiated T cells with concomitant loss of naïve T cell numbers in HCMV infected individuals has been reported in previous studies where absolute cell numbers were determined (50, 69–71).

The numbers of NK cells and different subsets have also been studied by others, the increase in NKG2C expression on NK cells in HCMV positive individuals has been reported in many studies (63, 64). In this cohort there were no significant differences with age or HCMV serostatus on the total numbers of NK cells or NKT cells, which is broadly supported by other studies which have enumerated NK cells, although one study did observe an increase in CD56dim NK cells with age (70), and another study saw a significant decrease in NKT numbers associated with HCMV positivity (69). We also observed an association of HCMV seropositivity with decreasing numbers of CD19+ B cells, which is unique to this cohort as two prior studies have not observed this effect associated with total B cell numbers (69, 70). Lastly, we also enumerated the numbers of monocytes present in our donor cohort, with the numbers of CD14 positive monocytes significantly increased in the HCMV positive donors. The importance, if any, of this observation is unclear except that monocytes are a known site of HCMV carriage in humans (8), however a large population study did not see an effect of HCMV on monocyte numbers but did see a significant decrease in plasmacytoid dendritic cells in older CMV positive donors (72). Measurement of lymphocyte numbers in the peripheral blood of the donor cohort is important for establishing that the recruited healthy donors have cell numbers within typical reference values, which was the case for this cohort.

To date our understanding of the cellular response to HCMV within an ageing population has been based on determining the magnitude of the HCMV specific T cell response, in many cases to one or two immunodominant proteins as either peptide pools or using MHC allele mapped epitopes. Although expanding the number of HCMV proteins surveyed for responses provides more information about the entire HCMV reactive T cell pool (73), it has been shown to have limited applicability to predicting protective immune responses (74). In the murine cytomegalovirus (MCMV) model system the phenomenon of memory inflation over time post infection has been well established. However, in contrast, there is limited evidence of inflation of the memory CMV specific responses in humans over time and with increasing age (23). In this donor cohort, measuring the cytokine responses to five HCMV protein pools of overlapping peptides, we did not observe an effect of age on the magnitude or breadth of the IFNγ, TNFα or IL-10 responses to HCMV peptide stimulation. Although we did see a significant increase in the magnitude of the CD8+ T cell IFNγ response to stimulation by the pp71 and US3 protein peptide mix in the older donors, this may be the effect of the smaller cohort as we did not observe any effect of age on the magnitude of the T cell response to any of the different HCMV proteins previously in a larger donor cohort (50). There also was no effect of age on the relationship between the magnitude of the summed IFNγ response with the anti-viral capacity of CD4+ and CD8+ T cells, there was no significant association between HCMV specific T cell responses and control of viral dissemination in either the young or old seropositive donors.

The use of overlapping peptide pools of HCMV proteins to assess T cell cytokine responses allows better enumeration and determination of the breadth of the anti-HCMV T cell responses but does not determine the effector function of these T cells against HCMV infected cells when the virus is expressing immune evasion proteins. T cell responses to HCMV infected dendritic cells (DCs) tend to be more polyfunctional than those elicited by peptide pool stimulation and others consider infected DCs as the best predicator of anti-HCMV T cell immunity (75). A viral dissemination assay was developed as a fully autologous system that allows the determination of cellular responses to HCMV infection *in vitro*, using this system we have established that it is possible to interrogate and compare the abilities of CD8+ T cell, CD4+ T cell and NK cells to control the spread and replication of HCMV (51–54). Our previous studies with this system have shown that while HCMV lysate stimulated PBMC secreted proteins have an anti-viral effect this varies between donors and depletion of IFNγ did not abrogate the anti-viral response (53). The ability of CD8+ T cells to control viral spread requires antigen presentation *via* MHC class I molecules as neutralizing this interaction results in increased viral dissemination. In addition to direct cytotoxic actions against infected cells, both CD8+ T cells and NK cells can control viral dissemination using non-cytotoxic mechanisms where granzymes are released into the infected cell and degrade IE proteins (76). Here we show there is an age-related defect in the ability of the adaptative cellular immune response to control viral dissemination, with total PBMCs, CD8+ T cells and CD4+ T cells from younger donors being significantly better at control of viral spread measured by early phase infection. However, it is also clear that the adaptative cellular response from the older donor cohort does show anti-viral control of HCMV compared to the seronegative donor cohort which is expected given that HCMV seronegatives have no HCMV antigen specific memory T cells. Deciphering which element of the complex system of secreted proteins and cell to cell interactions comprise the age-related defect we have observed will help to inform ongoing studies to develop new therapeutic interventions to prevent viral reactivation and the subsequent morbidity and mortality in vulnerable patient groups. The NK cells co-culture experiments revealed that there is no association with HCMV infection status and the ability of NK cells to control viral dissemination. We observed a range of NK cell effector function among the three donor cohort groups analyzed and the factors influencing the ability of NK cells from some donors to control viral dissemination in these assays while other do not warrants further investigation.

There is increasing evidence that the detrimental age associated effects of immune responses is associated with age related changes in the microenvironment where immune cells are recruited (77). This has been seen in the tumor microenvironment where the incidence of cancers increases and the ability of the immune system to control them decreases in older humans (78). Evidence from studies in humans of the immune response in the skin has shown that an increase in the number of senescent fibroblasts, identified by upregulation of non-canonical human leukocyte molecule HLA-E (67), leads to the recruitment of monocytes which inhibit the antigen specific T cell response to immune challenge by VZV skin tests (66). This confirmed earlier observations that there was no difference in the composition of VZV specific T cells between young and old donors isolated from the peripheral blood, however in the older donor’s skin there was an increase in the number of T regulatory cells and in PD-1 expression compared to the young donor skin resident VZV specific cells, suggesting that older donor skin resident T cells are functional and it is local environmental signals that may affect the responses observed (79).

There is also clear evidence from studies in humans and using aging mouse models that there are changes to stromal cells in the lymph node which effect the triggering of important recall immune responses due to defective location of memory T cells in the aged lymph node (80–82). This evidence suggested to us that the defect in the ability of HCMV specific T cells to control viral spread in our VDA system may be a result of increased expression of inhibitory ligands on the dermal fibroblasts and their corresponding receptors on the T cells. We have examined a range of inhibitory ligands on the dermal fibroblasts used in this cohort. We observed increased expression of PD-L1 and CD86 on the older donor dermal fibroblasts and significantly increased expression of B7-H3 and HLA-E. HCMV encodes a polymorphic glycoprotein UL40, which encodes a sequence that can bind HLA-E independently and effect the affinity of the interaction of HLA-E with the CD94 and NKG2C or NKG2A dimers, the effects of the different sequences can reduce HLA-E expression on the cell surface or reduce the affinity of HLA-E binding with the NKG2 receptor (83). Certain of these UL40 variants are associated with highly viremic episodes post lung transplant, due to better inhibition of the NKG2A+ NKG2C- NK cells (84). UL40 also induces an unconventional HLA-E restricted CD8+ T cell response (85), this population which also expresses multiple NK associated receptors may contribute to poorer outcomes post kidney transplantation (86). HLA-E expression has been observed to be increased on older human dermal fibroblasts identified in skin biopsy staining and blocking the interaction between NKG2A and HLA-E boosted immune responses in the skin (67). Senescent cells that express HLA-E are usually cleared by cytotoxic NK and CD8+ T cells *via* interaction with the activating NKG2C receptor and ligation of MICA/ULBP2 with the NKG2D receptor (77), however HLA-E also binds the inhibitory NKG2A receptor which may prevent cytotoxic activity of the HCMV specific T cell against the infected fibroblast. An analysis of NKG2 A, C and D expression levels on T cell and NK cells between our young and old donor cohorts would also be informative in understanding the balance between activating and inhibitor signaling that might be occurring through these important receptors. The receptor for PD-L1, PD-1, is known to be expressed by HCMV specific CD4+ T cells and is associated with a reduction in anti-viral cytokine production (87, 88). In sepsis patients HCMV reactivation is associated with upregulation of PD-1 expression on CD8+ T cells and a loss of polyfunctionality (89). The evidence from the literature of inhibitory receptor expression on HCMV specific T cells and our observations of inhibitory ligand expression on the dermal fibroblasts suggests that understanding which of these inhibitory ligand and receptor interactions are occurring within the AQUARIA cohort could provide an explanation for the age associated defect we observed. Further work investigating the impact of HCMV infection on the expression of inhibitory ligands on both infected and bystander cells, as well as characterizing the expression or not of inhibitory receptors on HCMV specific T cells is warranted. If these pathways prove to be involved in the loss of control of viral spread *in vitro* it opens the possibility of therapeutic intervention using the well-established immune checkpoint blockade reagents utilized in cancer treatment (90) to improve patient outcomes in the pathogenesis of HCMV and other infectious diseases (91).

Whilst the cellular immune response to HCMV is essential in controlling primary and reactivating infection (6), the humoral immune response is also likely to play an important role in the adaptative immune response and that a protective response to HCMV is heterogenous in nature, requiring cellular and humoral components (92). The humoral memory response to a reactivating viral infection is composed of protective antibodies secreted by plasma cells and also by antibodies secreted following reactivation of memory B cells populations (93). The first antibody produced in the anti HCMV humoral immune response are the low affinity IgM isotype (94) and following affinity maturation of antibodies the isotype switches to IgG and also IgA which is associated with the salivary mucosa (95).

The majority of diagnostic assays for HCMV measure the amount of IgG antibody present to antigens derived from virus cultured in fibroblasts or fibroblast lysate, often using the laboratory adapted strain AD169, such as is used by the commercial Captia EIA used in this study or alternatively recombinant CMV antigens to specific HCMV membrane associated proteins (96). Measurement of the total IgG present in the serum of the AQUARIA cohort reactive to HCMV antigens, as well as gB and the pentameric complex [gH/gL/pUL128-130-131 (68)] proteins did not reveal a significant difference between the amount of IgG in young donor serum compared to old donor serum, although there was a trend in all three assays to lower titers in the old donor cohort. However, measuring the total anti-HCMV serum IgG titers does not provide information about the HCMV neutralizing capacity of the humoral immune response in our donor cohort. In order to assess this, neutralization assays were performed on fibroblasts and epithelial (ARPE-19) cells to determine how effective the serum derived from these donors was at preventing infection. The use of the two different cell types provided useful information about the target of the neutralizing antibody response as it has been shown that the trimer complex of gH/gL/gO is required for HCMV entry into fibroblast cells and the pentamer complex of gH/gL/pUL128-130-131 is required for viral entry into endothelial and epithelial cells, both complexes are considered major targets of neutralizing antibodies (97). The older donors had significantly poorer neutralization of HCMV infection of epithelial cells compared to the young HCMV seropositive donor cohort, indicating that there is a defect in the memory response when it directly encounters the virus, which is not observed when measuring the total amount of HCMV specific IgG. Measurement of the avidity of the binding of the gB and pentamer specific IgG in the HCMV seropositive donors did not show any effect of age, indicating that the antibodies in older donors are not worse but they possibly undergone affinity maturation towards epitopes that are not required for neutralization.

While a defect in neutralization of HCMV infection has not been described in the context of aging, others have shown that in primary infection in pregnant women the development of neutralizing antibodies against the pentamer complex correlated with prevention of transmission to the fetus (68). However, in another study virus neutralization was not predictive of protection against HCMV reactivation in solid organ transplant recipients whereas T cell responses to HCMV infected dendritic cells were (98). While a defect in the functionality of antibodies produced by the memory B cell response to HCMV in the elderly has not been reported, there have been studies looking at the effect of age on HCMV B cell memory. The first study reported an increase in the memory B cell pool reactive to HCMV in older healthy volunteers and observed an increase in HCMV specific IgG plasma levels (99), which is the opposite of our observations. Analysis of the B cell heavy chain repertoire in young and old donors stratified by CMV serostatus observed a similar effect on diversity and memory populations as in T cells, where there is a decrease in naïve sequences and diversity with age which is also seen in CMV positive young donors (100). Exploring the functionality of neutralizing antibody responses against a clinical HCMV viral strain has revealed that whilst the older donors do have functional antibodies as they are able to neutralize infection compared to seronegative donors there is a loss in the quality of neutralization capacity of the antibodies produced possibly due to age related effects on antibody affinity maturation (101). It may also be due to long term carriage of the virus by older donors as the production of pentamer specific antibodies arises early during primary infection (102).

We have previously shown that detection of HCMV DNA in HCMV seropositive donors is rare event (1/41 donors aged >65 years) (50), suggesting that there may be a slight loss of control of viral replication in older CMV positive donors which does not result in overt disease. We hypothesized that if we looked at bodily fluids from other peripheral tissue sites such as urine and saliva in addition to whole blood we may be able to detect HCMV reactivation *in vivo* within the donor cohort. There have been previous studies in adults that suggest HCMV can be detected in both urine from older donors (47) and saliva from young and old donors (103, 104). We detected HCMV DNA in the saliva of 2/9 of the older HCMV positive donor cohort, but no HCMV DNA was detected in any of the young donor blood, urine or saliva samples. This confirms previous studies which have shown that there is increased detection of HCMV DNA in older donors which is absent in young (47, 48) and supports the idea that there is a loss of functional quality of the adaptative immune response to HCMV infection by the older donor cohort. Analysis of the quality of the immune response of the two older donors with HCMV DNA detectable in their saliva also supports this hypothesis, in that these two donors have decreased control of viral spread by their PBMC and CD4+ T cells at the early phase of infection. Interestingly, they have better control of viral dissemination by their NK cells compared to all the other donors examined, suggesting there may be an alteration in the balance between innate and adaptative immune response in resulting in HCMV reactivation at mucosal sites and detectable HCMV DNA.

Overall, we have shown that there is a defect in aspects of the adaptative cellular and humoral response to HCMV in older donors and this loss of functional control of HCMV correlates with evidence of increased viral replication at peripheral tissue sites in some of these donors. The resulting increased inflammation from viral reactivation may help to explain the reports of HCMV as a co-morbidity factor in the unwell aged including poorer outcomes in SARS-CoV2 infections (34, 105), increased mortality in older severe sepsis patients with HCMV reactivation (89) and the many associations of HCMV carriage with increased pathology in cardiovascular disease (35–40, 45, 46). The results from this study underline the importance of examining immune functions in response to active infections using clinical strains of HCMV *in vitro* as the interaction of the immune response with the virus reveals differences in the quality of the immune response in the old which merely measuring the magnitude of T cell and antibody responses to HCMV proteins alone did not. Using functional studies investigating immune cells ability to control viral spread and infection will also increase our understanding of how these processes occur in the body. Furthermore, they also demonstrate the importance of examining the antigen presenting cell (APC) – we clearly observe expression of some inhibitory ligands is increased on the dermal fibroblasts grown from the older donors. Through a functional assay that allows both the APC and immune cell to be interrogated in concert (e.g., the viral dissemination assay) it becomes possible to interrogate whether the interaction of these ligands with inhibitory receptors on the HCMV specific T cells plays a role in diminishing the ability of adaptative immune cells to control viral spread. If the interactions between these checkpoint inhibitory receptors and ligands are shown to play a role in causing the defect in the HCMV immune response in the old observed in this study, it provides a potential opportunity to therapeutically intervene to prevent HCMV reactivation using well established checkpoint inhibitor treatments.

## Data availability statement

The original contributions presented in the study are included in the article/**Supplementary Material**. Further inquiries can be directed to the corresponding authors.

## Ethics statement

The studies involving human participants were reviewed and approved by North of Scotland Research Ethics Committee 1 (NS/17/0110). The patients/participants provided their written informed consent to participate in this study.

## Author contributions

SJ, MR, and MW designed research. ED, MN, YL, CH, GO, CA, and SJ performed research. ED, MN, SJ, MR, and MW analyzed data. SJ and MW wrote the paper. All authors contributed to the article and approved the submitted version.
