# Supplementary material for: HCMV carriage in the elderly diminishes anti-viral functionality of the adaptive immune response resulting in virus replication at peripheral sites
Source: Front Immunol. 2022 Dec 15;13:1083230. doi: 10.3389/fimmu.2022.1083230 (PMC9797693; doi:10.3389/fimmu.2022.1083230)
Supplement: Supplementary file 1 [file DataSheet_1.pdf]

Supplementary Material

1 Supplementary Tables

Table S1: Absolute count antibody panel

Antigen	Fluorochrome	Clone	Isotype	Cat. No.	Supplier
CD19	BUV395	SJ25C1	IgG1 κ	563549	BD BioSciences
CD45	VioBlue	REA747	rh IgG1	130-110-637	Miltenyi Biotec
CD3	VioGreen	REA613	rh IgG1	130-113-142	Miltenyi Biotec
CD14	BV570	M5E2	IgG2a κ	301832	BioLegend
CD4	BV605	OKT4	IgG2b κ	317438	BioLegend
CD16	BV650	3G8	IgG1 κ	302042	BioLegend
CD56	BV785	5.1H11	IgG1 κ	362550	BioLegend
CD45RA	FITC	HI100	IgG2b κ	304106	BioLegend
CD8	PerCP-Cy5.5	HIT8a	IgG1 κ	300924	BioLegend
NKG2C	PE	REA205	rh IgG1	130-119-776	Miltenyi Biotec
CD57	PE-Dz594	HNK-1	IgM κ	359620	BioLegend
HLA-DR	PE-Cy5	L243	IgG2a κ	307608	BioLegend
CD127	PE-Cy7	A019D5	IgG1 κ	351320	BioLegend
CD25	APC	BC96	IgG1 κ	302610	BioLegend
CD28	AxF700	CD28.2	IgG1 κ	302920	BioLegend
CD27	APC-Cy7	O323	IgG1 κ	302816	BioLegend

Abbrv: BV = Brilliant Violet; BUV = Brilliant UV; Dz = Dazzle; AxF = Alexa Fluor

Table S2: qPCR Primers

		5’ MOD		3’ MOD
Sense Primer	gB1		GAG GAC AAC GAA ATC CTG TTG GGC A	
Anti-sense Primer	gB2		GTC GAC GGT GGA GAT ACT GCT GAG G	
Probe Sequence	P3	6FAM	CAA TCA TGC GTT TGA AGA GGT AGT CCA	BHQ1

Table S3: Fibroblast Inhibitory Flow cytometry panel

Antigen	Fluorochrome	Clone	Isotype	Cat. No.	Supplier	Panel
CD324 (E-cadherin)	BUV397	67A4	IgG1κ	743717	BD Biosciences	1 & 2
HLA ABC	BUV737	G46-2.6	IgG1κ	741844	BD Biosciences	1 & 2
CD45	BV570	HI30	IgG1κ	304034	BioLegend	1 & 2
CD36	BV605	CB38	IgM	563518	BD Biosciences	1 & 2
CD80 (B7-1)	BV650	2D10	IgG1κ	305227	BioLegend	1 & 2
CD31	BV785	WM59	IgG1κ	303148	BioLegend	1 & 2
HLA DR-DP-DQ	PerCP-Cy5.5	Tu39	IgG2aκ	361710	BioLegend	1 & 2
CD86 (B7-2)	PE-Cy5	IT2.2	IgG2bκ	305408	BioLegend	1 & 2
CD90 (Thy-1)	AxF700	5E10	IgG1κ	328120	BioLegend	1 & 2
CD39	APC-Cy7	A1	IgG1κ	328226	BioLegend	1 & 2
Galectin-9	BV421	9M1-3	IgG1κ	348920	BioLegend	1
PD-L2 (CD273)	BV421	24F.10C12	IgG2aκ	329616	BioLegend	2
ISOSL (B7-H2)	FITC	REA991	rhIgG	130-116-801	Miltenyi Biotec	1
GITRL	FITC	REA841	rhIgG	130-112-972	Miltenyi Biotec	2
HVEM (CD270)	PE	122	IgG1κ	318806	BioLegend	1
B7-H4	PE	MIH43	IgG1κ	358104	BioLegend	2
HLA-E	PE-Dz594	3D12	IgG1κ	342616	BioLegend	1
PD-L1 (CD274)	PE-Dz594	29E.2A3	IgG2bκ	329732	BioLegend	2
CD155 (PVR)	PE-Cy7	SK11.4	IgG1κ	337614	BioLegend	1
CD70 (CD27L)	PE-Cy7	113-16	IgG1κ	355112	BioLegend	2
B7-H3 (CD276)	APC	MIH42	IgG1κ	351006	BioLegend	1
VISTA	AxF647	MIH65	IgG1κ	566670	BioLegend	2
IgG1κ	BUV397	X40	Isotype	563547	BD Biosciences	1 & 2
IgG1κ	BUV737	X40	Isotype	612758	BD Biosciences	1 & 2
IgG1κ	BV570	MOPC-21	Isotype	400160	BioLegend	1 & 2
IgM	BV605	G155-228	Isotype	563517	BD Biosciences	1 & 2

IgG1κ	BV650	MOPC-21	Isotype	400164	BioLegend	1 & 2
IgG1κ	BV785	MOPC-21	Isotype	400170	BioLegend	1 & 2
IgG2aκ	PerCP-Cy5.5	MOPC-173	Isotype	400252	BioLegend	1 & 2
IgG2bκ	PE-Cy5	MPC-11	Isotype	400318	BioLegend	1 & 2
IgG1κ	AxF700	MOPC-21	Isotype	400144	BioLegend	1 & 2
IgG1κ	APC-Cy7	MOPC-21	Isotype	400128	BioLegend	1 & 2
IgG1κ	BV421	MOPC-21	Isotype	400158	BioLegend	1
IgG2aκ	BV421	MOPC-173	Isotype	400260	BioLegend	2
rhIgG	FITC	REA293	Isotype	130-113-437	Miltenyi Biotec	1 & 2
IgG1κ	PE	MOPC-21	Isotype	400114	BioLegend	1 & 2
IgG1κ	PE-Dz594	MOPC-21	Isotype	400176	BioLegend	1
IgG2bκ	PE-Dz594	MPC-11	Isotype	400358	BioLegend	2
IgG1κ	PE-Cy7	MOPC-21	Isotype	400126	BioLegend	1 & 2
IgG1κ	APC	MOPC-21	Isotype	400122	BioLegend	1
IgG1κ	AxF647	MOPC-21	Isotype	565571	BD Biosciences	2

Abbrv: BV = Brilliant Violet; BUV = Brilliant UV; Dz = Dazzle; AxF = Alexa Fluor

Table S4: Summarized mean positive responses to each HCMV protein mix

	**CD8+ T cells Positive sfu/CD3 10^6 Geomean (95% CI)**			**CD4+ T cells Positive sfu/CD3 10^6 Geomean (95% CI)**		
	**IFNγ**	**TNFα**	**IL-10**	**IFNγ**	**TNFα**	**IL-10**
**LAT**	1508 (793, 2866)	1492 (348, 6401)	1000 (327, 3057)	892 (447, 1781)	2159 (678, 6872)	586 (291, 1181)
**pp65 UL144**	2659 (1135, 6229)	1220 (360, 4138)	588	1483 (782, 2813)	1317 (522, 3321)	315
**IEs**	1432 (600, 3415)	1956 (544, 7034)	339	423 (224, 800)	1145 (267, 4917)	105
**pp71 US3**	1017 (420, 2462)	2724 (647, 11461)	2840 (1660, 4859)	548 (270, 1113)	3652 (1354, 9853)	2274 (202, 25601)
**gB**	750 (370, 1526)	1220 (342, 4346)	430	1769 (727, 4301)	2390 (547, 10452)	135

2 Supplementary Figures

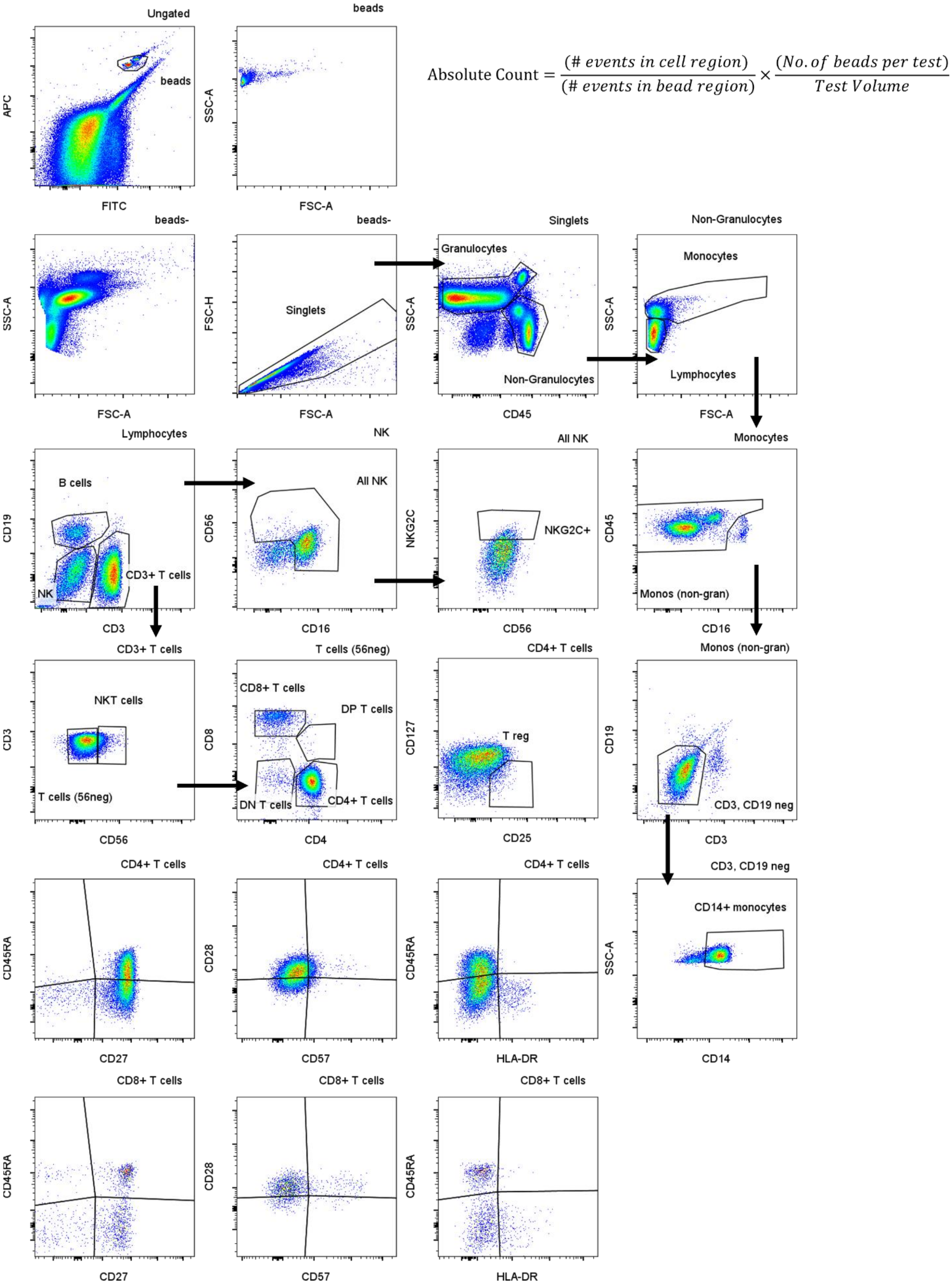

$$\text{Absolute Count} = \frac{(\#\ events\ in\ cell\ region)}{(\#\ events\ in\ bead\ region)} \times \frac{(No.of\ beads\ per\ test)}{Test\ Volume}$$

Supplementary Figure S1 – Absolute Count gating strategy and calculation

Representative dot plots from 1 donor are shown illustrating the gating strategy for generating the absolute count data. First the trucount bead population was identified (FITC vs APC) and then the trucount bead negative population (i.e. cells) were analysed by gating for single cells (FSC-A vs FSC-H), then CD45hi lymphocytes (Non-Granulocytes) and Granulocytes were gated (CD45 vs SSC-A). Next, a Monocytes gate and Lymphocytes gate were discriminated by size and granularity (FSC-A vs SSC-A) and monocytes were gated by eliminating CD16^{hi} cells (monos (non-gran gate on CD16 vs CD45 plot), the CD3 and CD19 negatives (CD3 vs CD19 plot) and finally the CD14+ monocyte population identified (CD14 vs SSC-A). From the lymphocytes gate CD3+ T cells, NK cells and B cells were identified (CD3 vs CD19 plot), the NK cells were then gated for CD56+, CD56+CD16+ and CD56-CD16+ populations as All NK (CD16 vs CD56) and the proportion of NK cells expressing NKG2C was enumerated (CD56 vs NKG2C). The CD3+ T cells were gated from the NKT cells (CD56+ by CD56 vs CD3 plot), then the CD4+ and CD8+ expressing cells and double positive (DP) T cells and double negative (DN) T cells were identified. T regulatory (Treg) CD4+ T cells were identified as CD127^{lo} and CD25^{hi} (CD25 vs CD127 plot). Lastly both the CD4+ and CD8+ T cell populations were further subdivided into 4 memory populations defined by expression of CD27 and CD45RA [T_{NAIVE} CD27+ CD45RA+; T_{CM} CD27+ CD45RA-; T_{EM} CD27- CD45RA-; T_{EMRA} CD27- CD45RA+], 4 differentiation populations defined by expression of CD57 and CD28 [Low Differentiation (Diff.) CD57- CD28+; Intermediate (Int.) Low Diff. CD57+ CD28+; Int. High Diff. CD57- CD28-; High Diff. CD57+ CD28-) and HLA-DR expressing activated cells were identified (HLA-DR vs CD45RA plot). All gate and quadrant positions were identified using the FMO controls, the formula used to calculate the absolute cell counts from the event numbers in each gate or quadrant is illustrated.

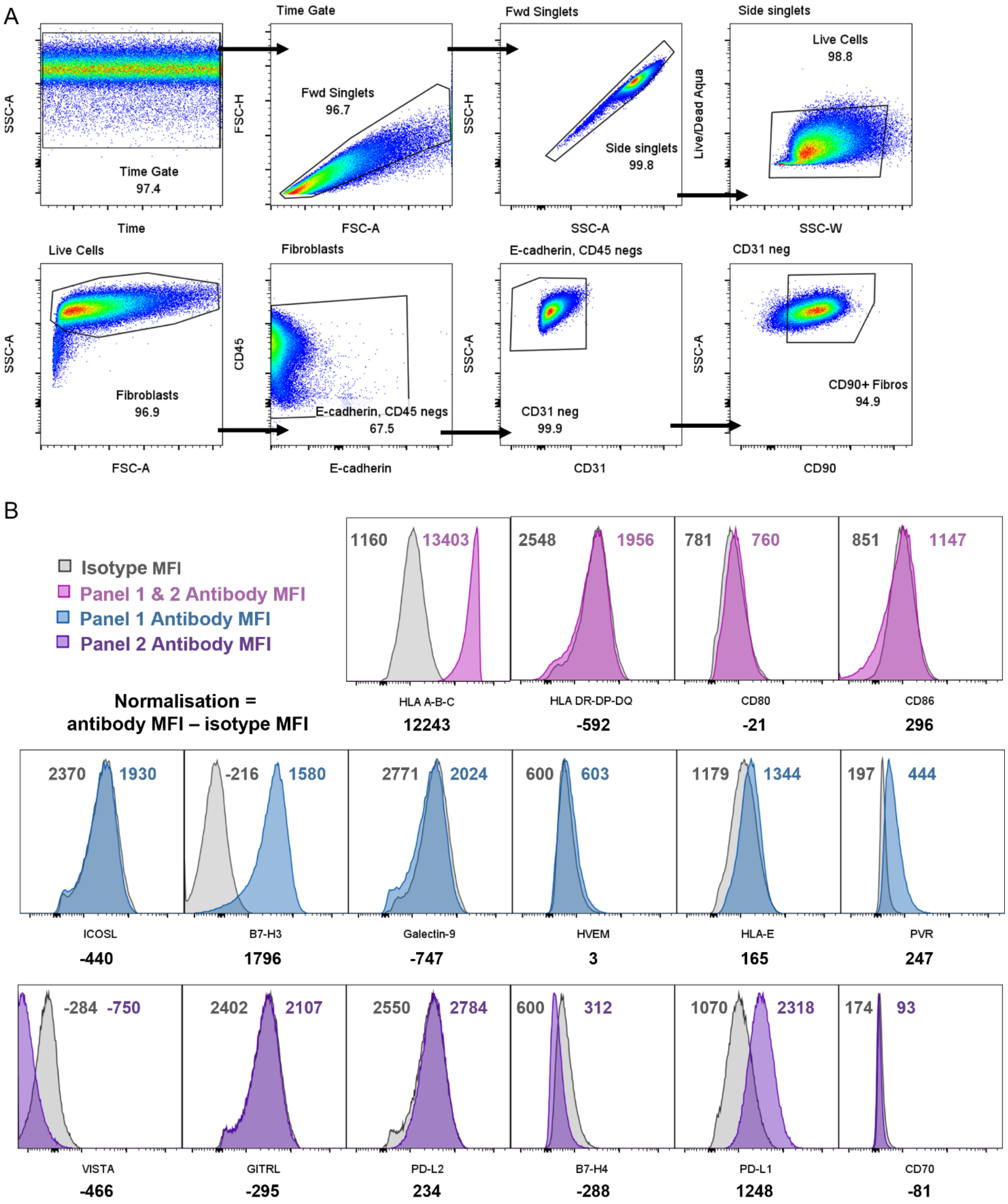
A
Time Gate
97.4
SSC-A
Time
Time Gate
Fwd Singlets
96.7
FSC-H
FSC-A
Fwd Singlets
Side singlets
99.8
SSC-H
SSC-A
Side singlets
Live Cells
98.8
Live/Dead Aqua
SSC-W
Live Cells
Fibroblasts
96.9
SSC-A
FSC-A
Fibroblasts
E-cadherin, CD45 negs
67.5
CD45
E-cadherin
E-cadherin, CD45 negs
CD31 neg
99.9
SSC-A
CD31
CD31 neg
CD90+ Fibros
94.9
SSC-A
CD90
B
Isotype MFI
Panel 1 & 2 Antibody MFI
Panel 1 Antibody MFI
Panel 2 Antibody MFI
Normalisation =
antibody MFI – isotype MFI
1160 13403
HLA A-B-C
12243
2548 1956
HLA DR-DP-DQ
-592
781 760
CD80
-21
851 1147
CD86
296
2370 1930
ICOSL
-440
-216 1580
B7-H3
1796
2771 2024
Galectin-9
-747
600 603
HVEM
3
1179 1344
HLA-E
165
197 444
PVR
247
-284 -750
VISTA
-466
2402 2107
GITRL
-295
2550 2784
PD-L2
234
600 312
B7-H4
-288
1070 2318
PD-L1
1248
174 93
CD70
-81

Supplementary Figure S2 – Fibroblast Inhibitory Ligand Flow cytometry gating strategy and expression calculation.

Representative dot plots from 1 donor are shown illustrating the gating strategy for gating the fibroblasts for analysis (A). First a Time vs Side scatter gate was drawn, to identify the main flow of cells, then these cells were gated for forward scatter single cells (Forward scatter area (FSC-A) vs Forward scatter height (FSC-H)), side scatter single cells (Side scatter area (SSC-A) vs Side scatter height (SSC-H)), live cells (SSC-W vs Live/Dead Aqua dye), then fibroblasts were gated (FSC-A vs SSC-A (log scale)). Fibroblasts were further identified using the lineage negative markers, first an E-cadherin and CD45 negative gate was drawn (E-cadherin (CD324) BUV397 vs CD45 BV570) and then CD31 negative cells were gated (CD31 BV785 vs SSC-A). Lastly, CD90+ fibroblasts were identified (CD90 AxF700 vs SSC-A) (1). Expression of the 16 different markers was assessed by measuring the geomean fluorescence intensity (MFI) on the fibroblasts (B) illustrated here as individual histograms overlayed on the respective isotype control for each antibody. Expression of each marker was normalized by subtracting the Isotype MFI from the specific antibody MFI. All MFI values are shown for each marker.

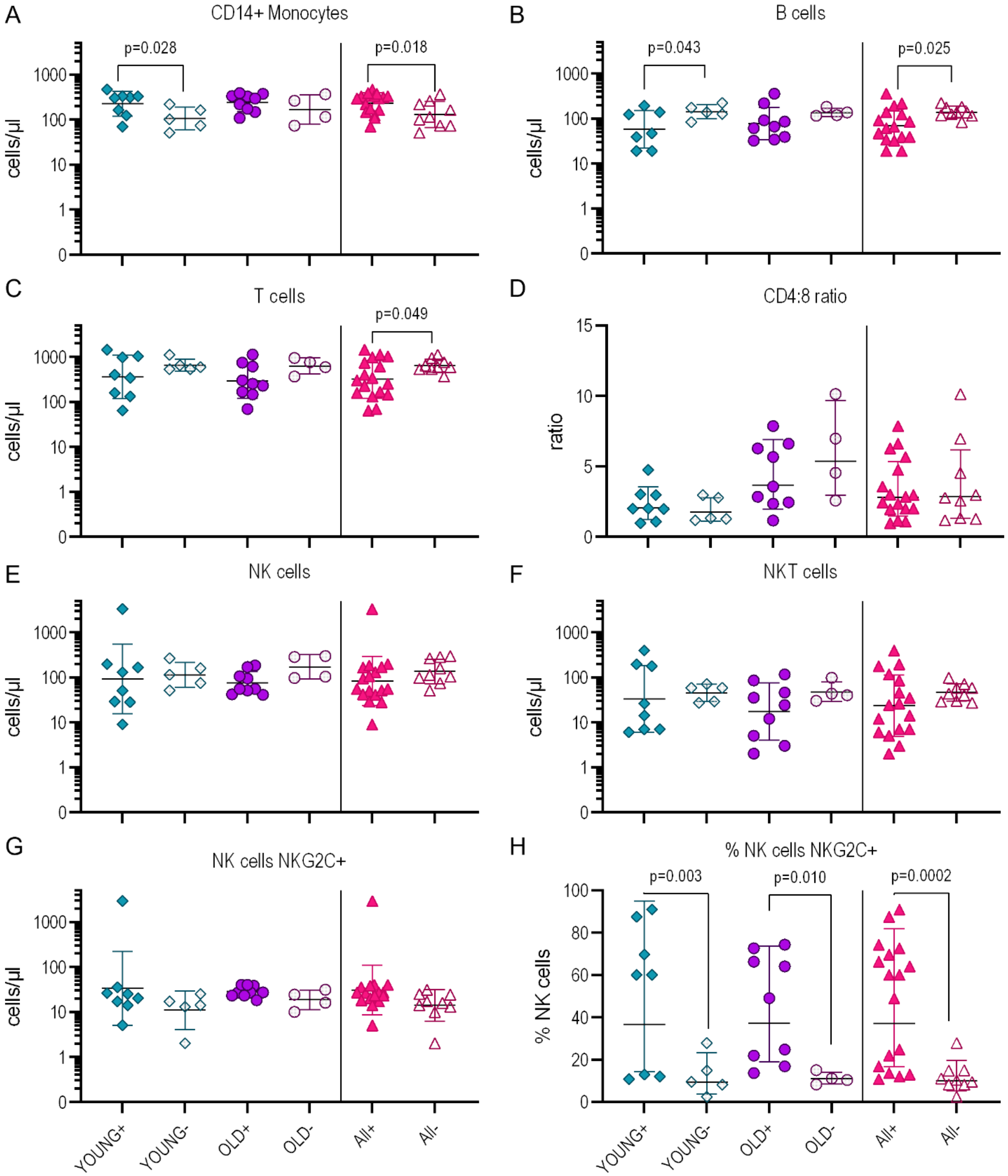
A
CD14+ Monocytes
p=0.028
p=0.018
cells/µl
B
B cells
p=0.043
p=0.025
cells/µl
C
T cells
p=0.049
cells/µl
D
CD4:8 ratio
ratio
E
NK cells
cells/µl
F
NKT cells
cells/µl
G
NK cells NKG2C+
cells/µl
H
% NK cells NKG2C+
p=0.003
p=0.010
p=0.0002
% NK cells
YOUNG+
YOUNG-
OLD+
OLD-
All+
All-

Supplementary Figure S3 – Absolute counts for Monocytes, B cells, total T cells and NK cells in the AQUARIA cohort

EDTA treated whole blood was stained with the panel of phenotyping antibodies in order to enumerate the different immune cell subsets as described in Figure S1. The number of cells per μl of whole blood from Young HCMV seropositive (YOUNG+), Young HCMV seronegative (YOUNG-), Old HCMV seropositive (OLD+), Old HCMV seronegative (OLD-) and the entire cohort HCMV seropositive (All+) and seronegative (All-) were compared. The numbers of monocytes (CD14+) (A), B cells (CD3- CD19+) (B), T cells (CD3+ CD19-) (C), the CD4:CD8 ratio (D), NK cells (CD3- CD19- then CD56+ CD16+/-) (E), NKT cells (CD3+ CD56+) (F) and NKG2C+ NK cells (G) and the proportion of NK cells NKG2C+ (H). The data is presented as scatter dot plots showing the geomean and geometric standard deviation for each group on each graph. The absolute count data was transformed and each cellular subset analysed by ordinary 1-way ANOVA with post-hoc Fisher's LSD test to compare between young and old groups and HCMV serostatus groups. Significant differences between groups are marked on the appropriate graph with the p-value indicated.

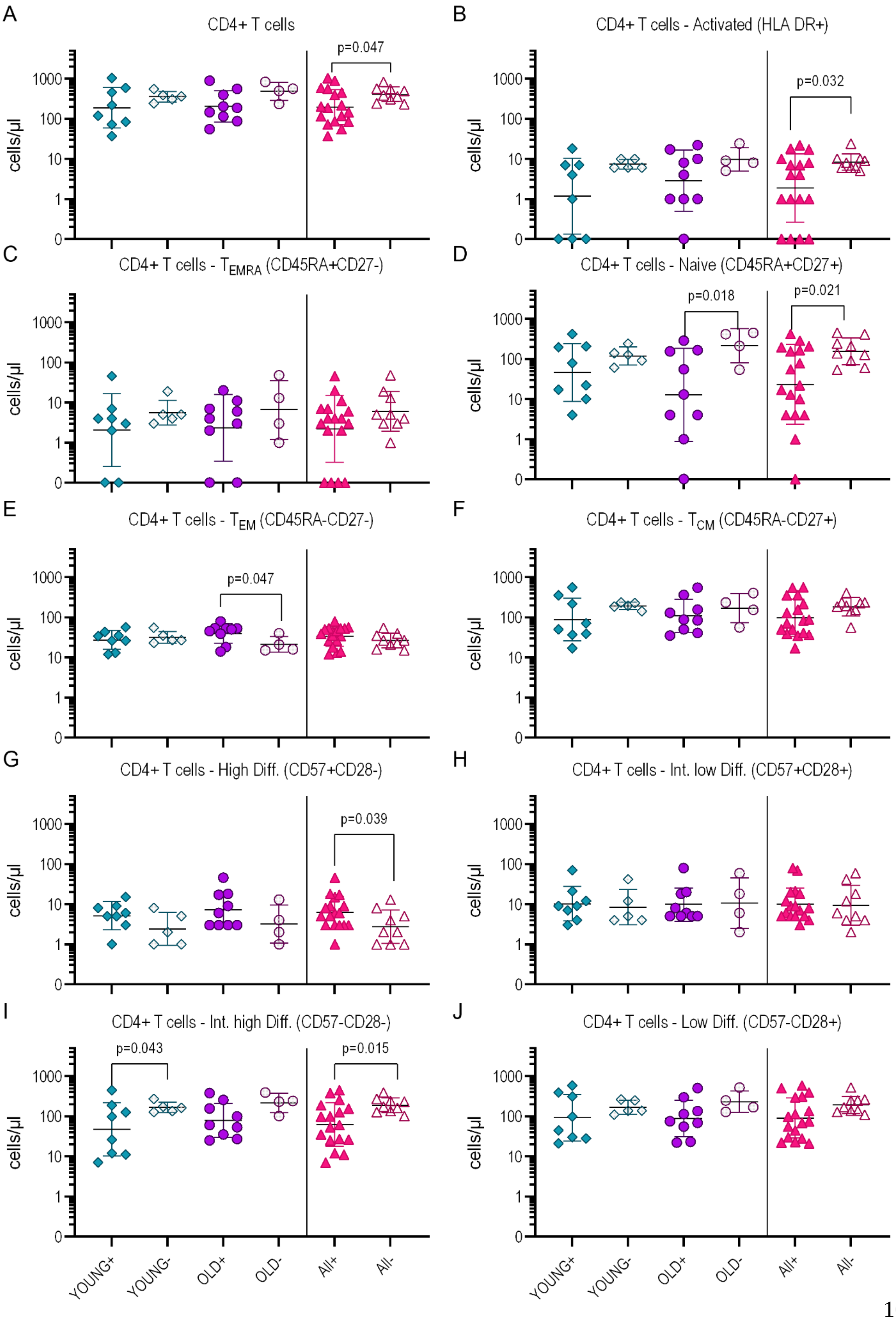
A
CD4+ T cells
p=0.047
B
CD4+ T cells - Activated (HLA DR+)
p=0.032
C
CD4+ T cells - TEMRA (CD45RA+CD27-)
D
CD4+ T cells - Naive (CD45RA+CD27+)
p=0.018
p=0.021
E
CD4+ T cells - TEM (CD45RA-CD27-)
p=0.047
F
CD4+ T cells - TCM (CD45RA-CD27+)
G
CD4+ T cells - High Diff. (CD57+CD28-)
p=0.039
H
CD4+ T cells - Int. low Diff. (CD57+CD28+)
I
CD4+ T cells - Int. high Diff. (CD57-CD28-)
p=0.043
p=0.015
J
CD4+ T cells - Low Diff. (CD57-CD28+)
cells/µl
1000
100
10
1
0
YOUNG+
YOUNG-
OLD+
OLD-
All+
All-

Supplementary Figure S4 – Absolute counts for CD4+ T cells and memory subsets

EDTA treated whole blood was stained with the panel of phenotyping antibodies to enumerate the numbers of CD4+ T cells and memory and differentiation subsets (illustrated in Figure S1). The number of cells per µl of whole blood from Young HCMV seropositive (YOUNG+), Young HCMV seronegative (YOUNG-), Old HCMV seropositive (OLD+), Old HCMV seronegative (OLD-) and the entire cohort HCMV seropositive (All+) and seronegative (All-) were compared. The numbers of total CD4+ T cells (CD8- CD4+) (A), Activated CD4+ T cells (CD4+ HLA-DR+) (B), Memory cell subsets defined by expression of CD27 and CD45RA, CD45RA re-expressing effector memory T cells (T_{EMRA} CD45RA+ CD27-) (C), Naïve T cells (T_{NAIVE} CD45RA+ CD27+) (D), Effector Memory (T_{EM} CD45RA- CD27-) (E) and Central Memory (T_{CM} CD45RA- CD27+) (F) were measured. CD4+ T cell differentiation subsets defined by expression of CD28 and CD57 (2) including highly differentiated CD4+ T cells (High Diff. CD57+ CD28-) (G), intermediate low differentiated CD4+ T cells (Int. low Diff. CD57+ CD28+) (H), intermediate highly differentiated CD4+ T cells (Int. High Diff. CD57- CD28-) (I) and least differentiated CD4+ T cells (Low Diff. CD57- CD28+) (J) were enumerated. The data is presented as scatter dot plots showing the geomean and geometric standard deviation for each group on each graph. The absolute count data was transformed and each cellular subset analysed by ordinary 1-way ANOVA with post-hoc Fisher's LSD test to compare between young and old groups and HCMV serostatus groups. Significant differences between groups are marked on the appropriate graph with the p-value indicated.

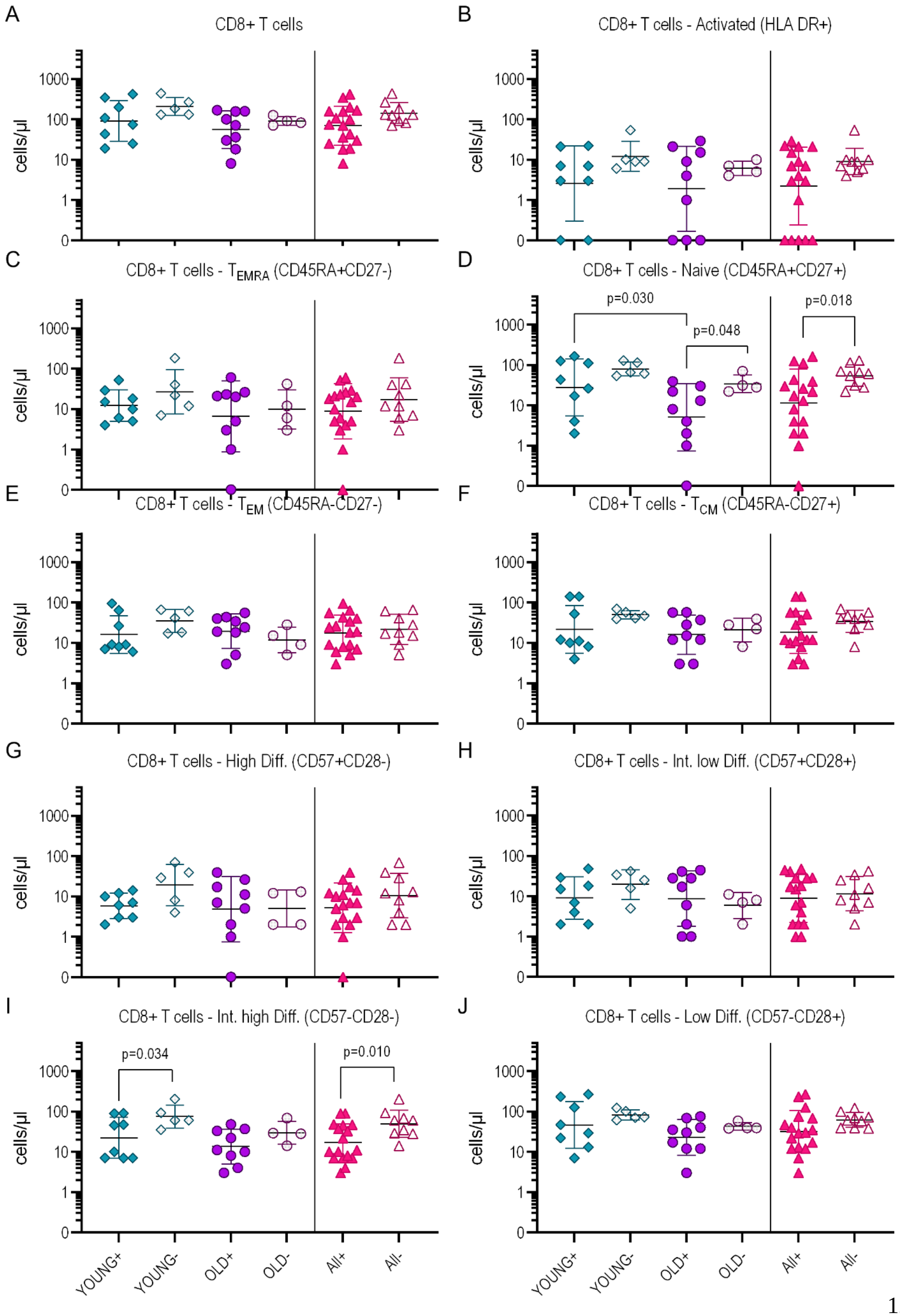
A
CD8+ T cells
B
CD8+ T cells - Activated (HLA DR+)
C
CD8+ T cells - T_EMRA (CD45RA+CD27-)
D
CD8+ T cells - Naive (CD45RA+CD27+)
p=0.030
p=0.048
p=0.018
E
CD8+ T cells - T_EM (CD45RA-CD27-)
F
CD8+ T cells - T_CM (CD45RA-CD27+)
G
CD8+ T cells - High Diff. (CD57+CD28-)
H
CD8+ T cells - Int. low Diff. (CD57+CD28+)
I
CD8+ T cells - Int. high Diff. (CD57-CD28-)
p=0.034
p=0.010
J
CD8+ T cells - Low Diff. (CD57-CD28+)
cells/µl
1000
100
10
1
0
YOUNG+
YOUNG-
OLD+
OLD-
All+
All-

Supplementary Figure S5 – Absolute counts for CD8+ T cells and memory subsets

EDTA treated whole blood was stained with the panel of phenotyping antibodies to enumerate the numbers of CD8+ T cells and memory and differentiation subsets (illustrated in Figure S1). The number of cells per µl of whole blood from Young CMV seropositive (YOUNG+), Young HCMV seronegative (YOUNG-), Old HCMV seropositive (OLD+), Old HCMV seronegative (OLD-) and the entire cohort HCMV seropositive (All+) and seronegative (All-) were compared. The numbers of total CD8+ T cells (CD8+ CD4-) (A), Activated CD8+ T cells (CD8+ HLA-DR+) (B), Memory cell subsets defined by expression of CD27 and CD45RA, CD45RA re-expressing effector memory T cells (T_{EMRA} CD45RA+ CD27-) (C), Naïve T cells (T_{NAIVE} CD45RA+ CD27+) (D), Effector Memory (T_{EM} CD45RA- CD27-) (E) and Central Memory (T_{CM} CD45RA- CD27+) (F) were measured. CD8+ T cell differentiation subsets defined by expression of CD28 and CD57 (2) including highly differentiated CD8+ T cells (High Diff. CD57+ CD28-) (G), intermediate low differentiated CD8+ T cells (Int. low Diff. CD57+ CD28+) (H), intermediate highly differentiated CD8+ T cells (Int. High Diff. CD57- CD28-) (I) and least differentiated CD8+ T cells (Low Diff. CD57- CD28+) (J) were enumerated. The data is presented as scatter dot plots showing the geomean and geometric standard deviation for each group on each graph. The absolute count data was transformed and each cellular subset analysed by ordinary 1-way ANOVA with post-hoc Fisher's LSD test to compare between young and old groups and HCMV serostatus groups. Significant differences between groups are marked on the appropriate graph with the p-value indicated.

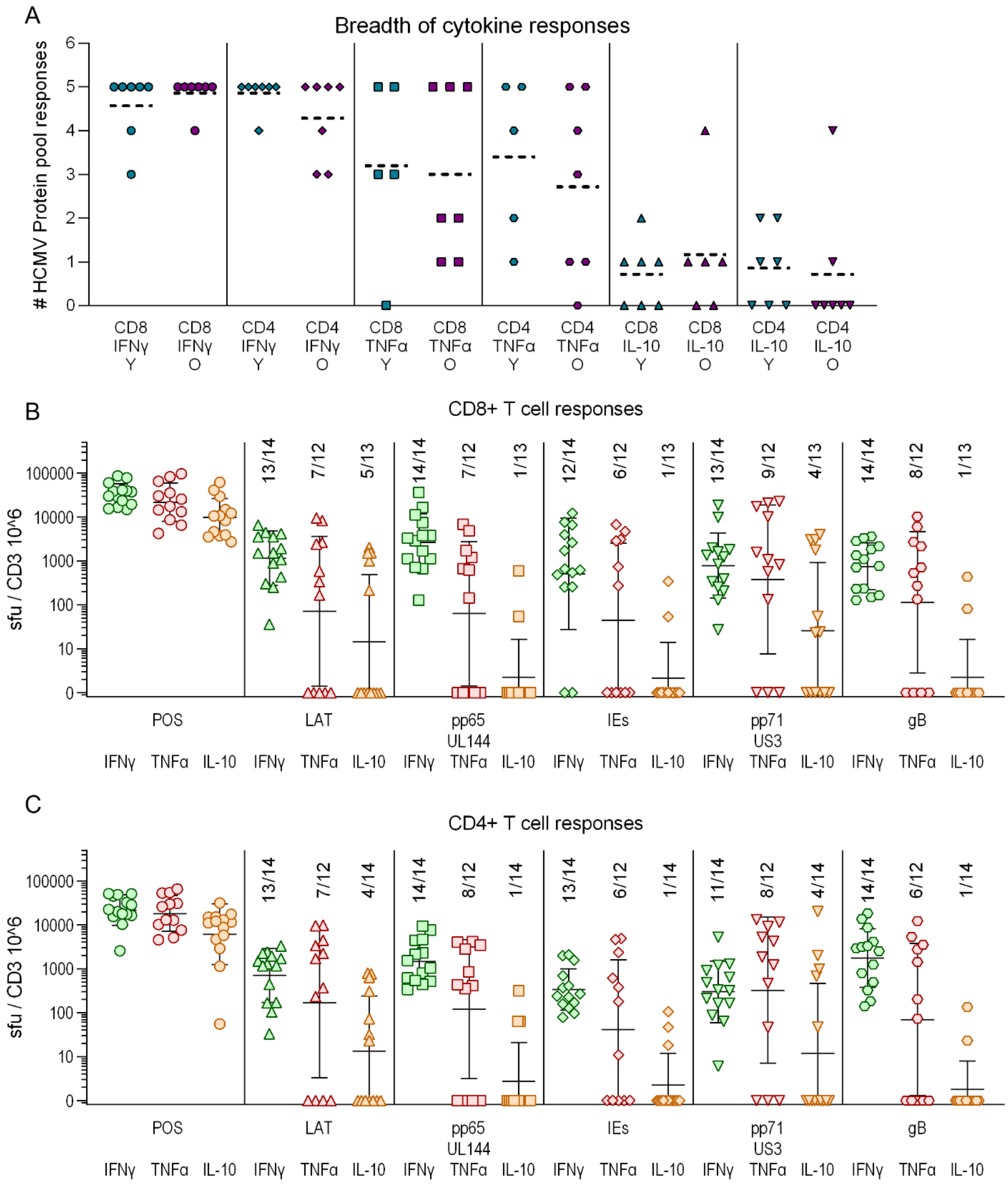

Supplementary Figure S6 – Breadth and donor frequency of HCMV specific T cell responses

The breadth of the three cytokines response to the 5 different HCMV protein mixes Latency associated proteins (LAT: UL138, US28, LUNA, vIL-10), pp65 and UL144, IE1 and IE2 (IEs), pp71 and US3 and gB proteins in the Aquaria donor cohort are illustrated (A), the breadth was calculated by summing the number of HCMV proteins with an above threshold cytokine (100 sfu/ million CD3+ T cells) response for each donor the mean of the young and old responses is shown as a dashed

line. All donors irrespective of age had a CD8+ and CD4+ T cell IFNγ response to a minimum of 3 of the HCMV protein mixes, with the majority of both old and young donors responding to all 5 HCMV protein mixes. The frequency of donors responding to the 5 different HCMV protein mixes for all three cytokines are illustrated for CD8+ T cells (B) and CD4+ T cells (C). The data is presented as scatter dot plots showing the geomean and geometric standard deviation for each age group on both graphs. All donors made a CD8+ and CD4+ T cell IFNγ response to pp65 and UL144 and gB stimulation, the highest frequency TNFα response was to pp71 and US3 stimulation in both T cell subsets, and stimulation by the latency associated proteins and also pp71 and US3 produced an IL-10 responses in CD8+ and CD4+ T cells.

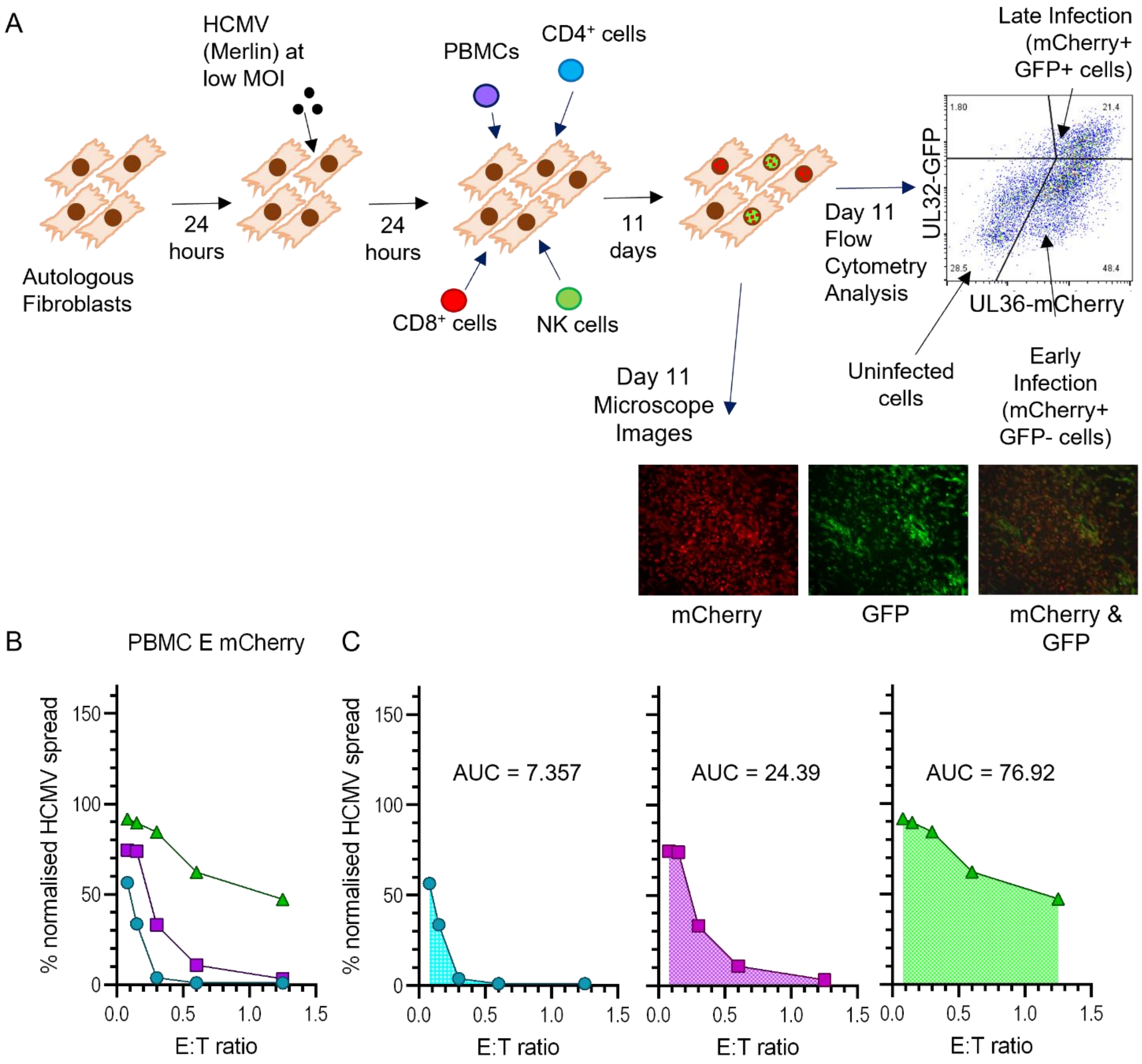

Supplementary Figure S7 – Viral Dissemination assay and Area Under the Curve (AUC) calculations.

The protocol for the viral dissemination assay are shown (A), 96 half-well plates were seeded with dermal fibroblasts and then infected with the dual fluorescence tagged strain of Merlin (UL36-mCherry and UL32-GFP) virus at a low multiplicity of infection. Twenty-four hours post infection autologous immune cell subsets are added at a range of effector to target (E:T) ratios and then co-cultured with the fibroblasts for 11 days. Fibroblasts were harvested and the mCherry and GFP expression was measured by flow cytometry with typical results at day 11 observed by flow cytometry and microscopy shown, illustrating immediate early gene expression of UL36 (mCherry+GFP- fibroblasts) indicative of viral spread and entry into cells and late gene expression of UL32 (mCherry+GFP+ fibroblasts) indicative that viral DNA replication has occurred; a full time course of infection with this virus is illustrated in figure 2 of Houldcroft *et al.* 2020 (3) showing the kinetics of expression of mCherry and then GFP over a 10 day time course. Viral spread in each well

was determined as a percentage of control infected wells without effector cells and control uninfected well to determine background fluorescence. The normalized viral spread data was plotted for all the E:T ratios and representative curves for early phase infection present in PBMC co-culture for a young CMV seropositive (turquoise circles), old CMV seropositive (purple squares) and CMV seronegative (green triangles) are shown (B). To allow comparison of viral control between individual donors, area under the curve calculations were performed and the calculated area is shown in part C (shaded area and value on each graph). Increasing AUC values reflects decreasing control of viral spread as shown by the seronegative (green shaded area) donor AUC value compared to the seropositive donor AUC values (turquoise and purple shaded areas).

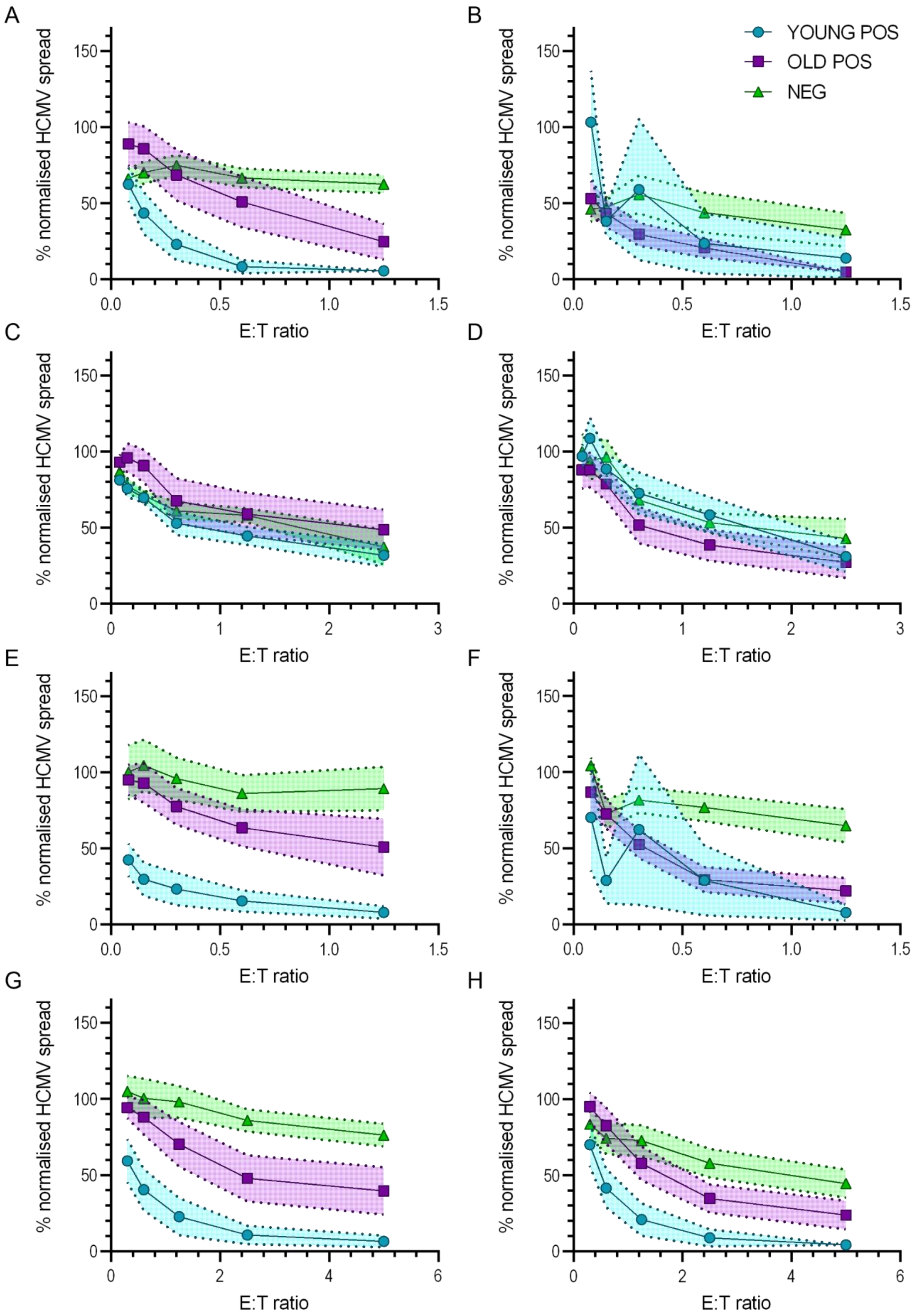
A
B
C
D
E
F
G
H
YOUNG POS
OLD POS
NEG
% normalised HCMV spread
E:T ratio
0
50
100
150
0.0
0.5
1.0
1.5
1
2
3
4
6

Supplementary Figure S8 – Anti-viral activity of PBMC, NK cells, CD8+ T cells and CD4+ T cells from Young and Old Seropositive and Seronegative donors

Ex vivo donor cells over a range of effector:target (E:T) ratios were co-cultured with autologous dermal fibroblasts infected with the dual fluorescence tagged Merlin strain of HCMV. After 11 days the cultures were harvested and analysed for mCherry and GFP expression by flow cytometry. Shown are the mean +/- standard error of the mean of the normalized viral dissemination for the three cohorts analysed, young positive n=6 (turquoise points and shading), old positive n=9 (purple points and shading) and seronegative donors n=9 (green points and shading) for both Early (mCherry+) gene expression (LH graphs) and late (GFP+ and mCherry+) gene expression (RH graphs). The results for PBMC (Early (A) and Late (B)), NK cells (Early (C) and Late (D)), CD8+ T cells (Early (E) and Late (F)) and CD4+ T cells (Early (G) and Late (H)) are shown. This clearly illustrates that PBMC, CD8+ and CD4+ T cells from the young donor cohort are more effective at controlling viral spread at Early gene expression time points compared to the old donor and seronegative donor cohorts.

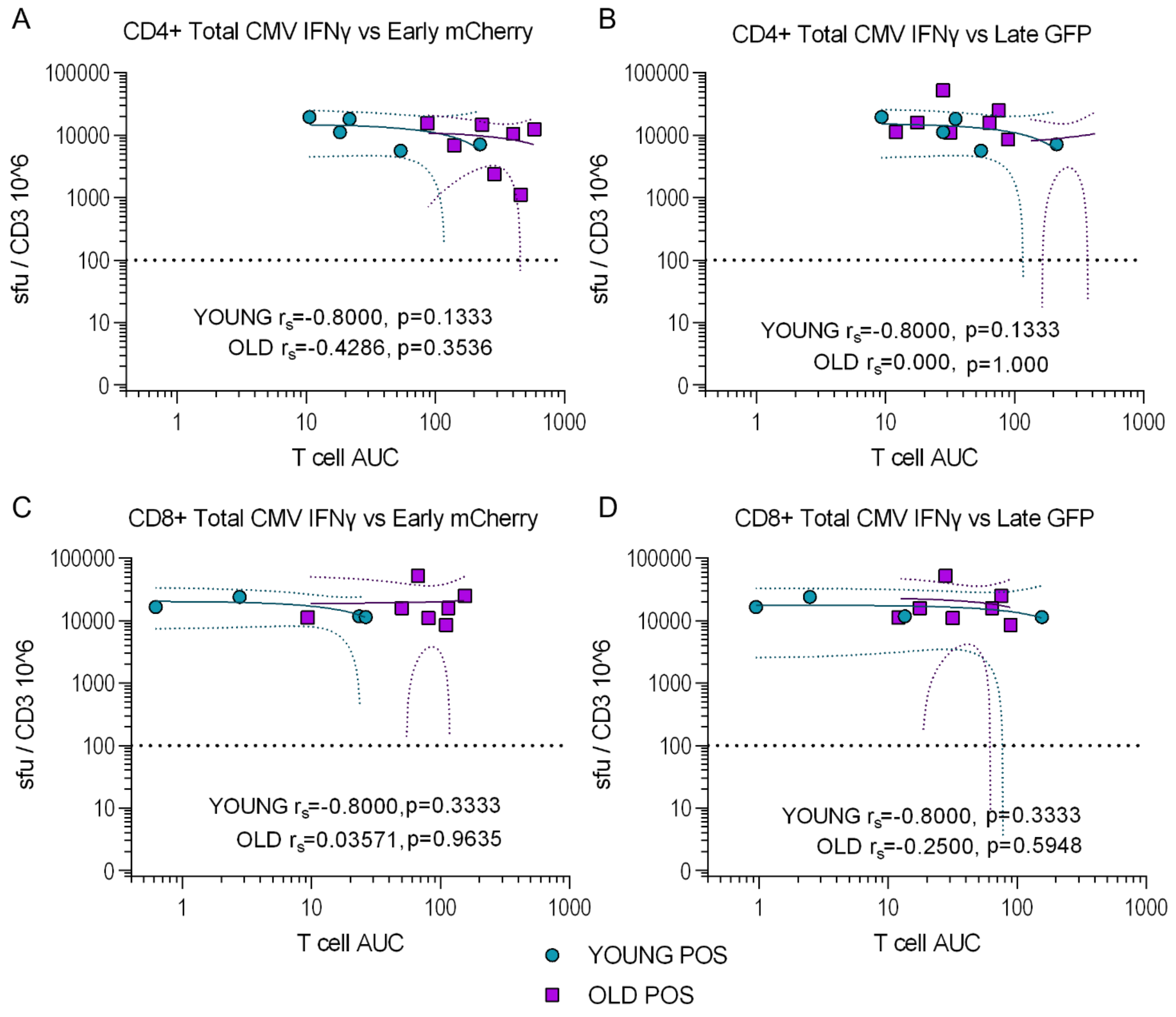

Supplementary Figure S9 – Correlation of VDA anti-viral activity with HCMV specific T cell responses

The anti-viral activity measure using the VDA (AUC values) of CD4+ (A & B) and CD8+ T cells (C & D) were correlated with the total HCMV specific IFNγ response (summed sfu/1x10^6 CD3+ T cells response from the 5 protein pool stimulations) for young (turquoise circles) and old (purple squares) seropositive donors for both Early and Late gene expression. The correlation of the HCMV specific T cell response with anti-viral activity of the individual T cell subsets was analysed using Spearman rank correlation (Spearman r_s and p value for young and old groups are indicated with the line of best fit (solid) and 95% CI (dotted lines) also shown on the graphs (Young turquoise lines and old purple lines)). Overall, this analysis shows that there is no correlation between the magnitude of the HCMV specific IFNγ secreting T cell response and the anti-viral activity in the viral dissemination assay.

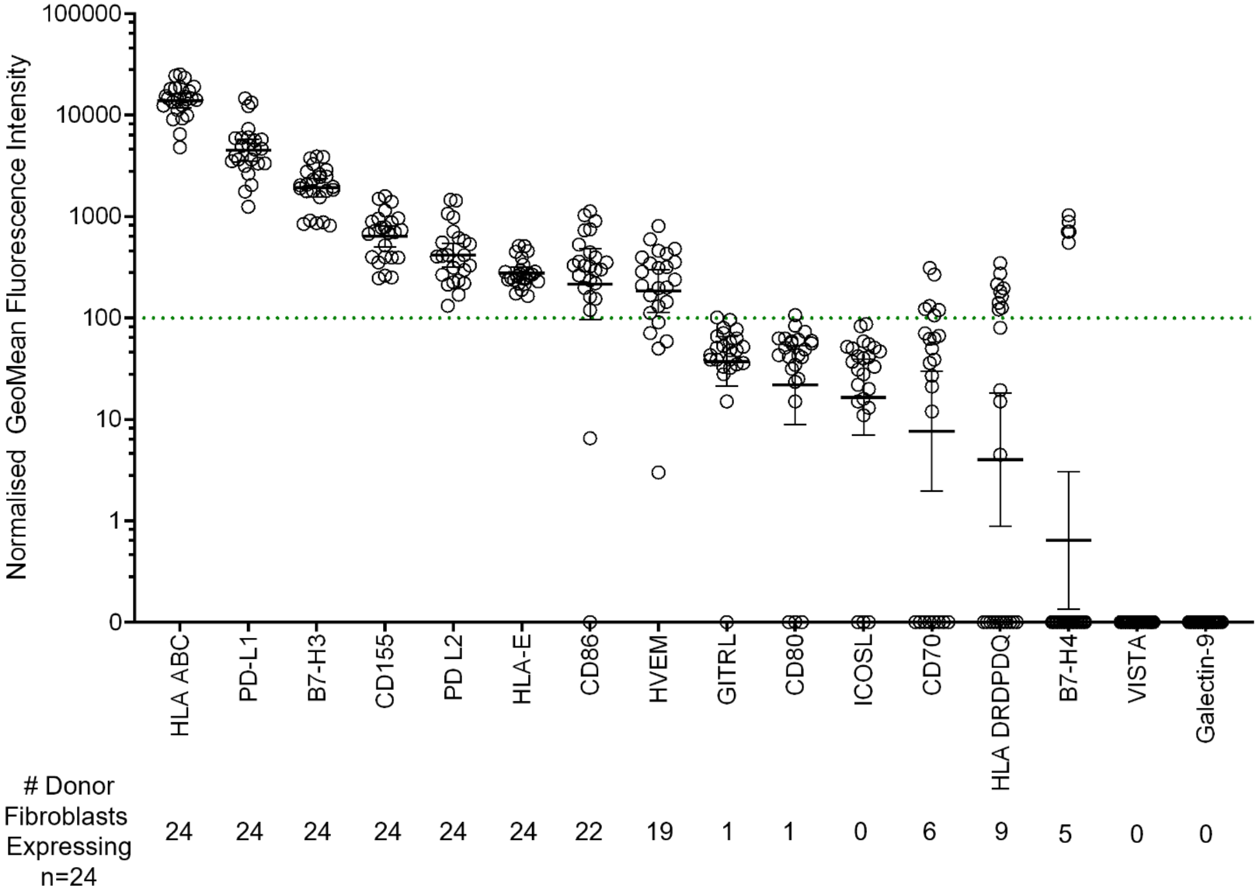

Supplementary Figure S10 – Expression of Inhibitory Ligand molecules on Dermal Fibroblasts

Expression of the ligands on dermal fibroblasts were normalized using the matching isotype controls (4), generating a normalized geomean fluorescence intensity(gMFI). The normalized gMFI expression of all sixteen molecules analysed are shown, the ligands with the highest expression (measured by the geomean of the normalised gMFI of the cohort (indicated along with the 95% Confidence Intervals of the data)) on the left with decreasing expression (geomean gMFI) to the right. Also shown are the number of the individual donor fibroblasts that have positive expression (above 100 units – marked as green dotted line) of each ligand.

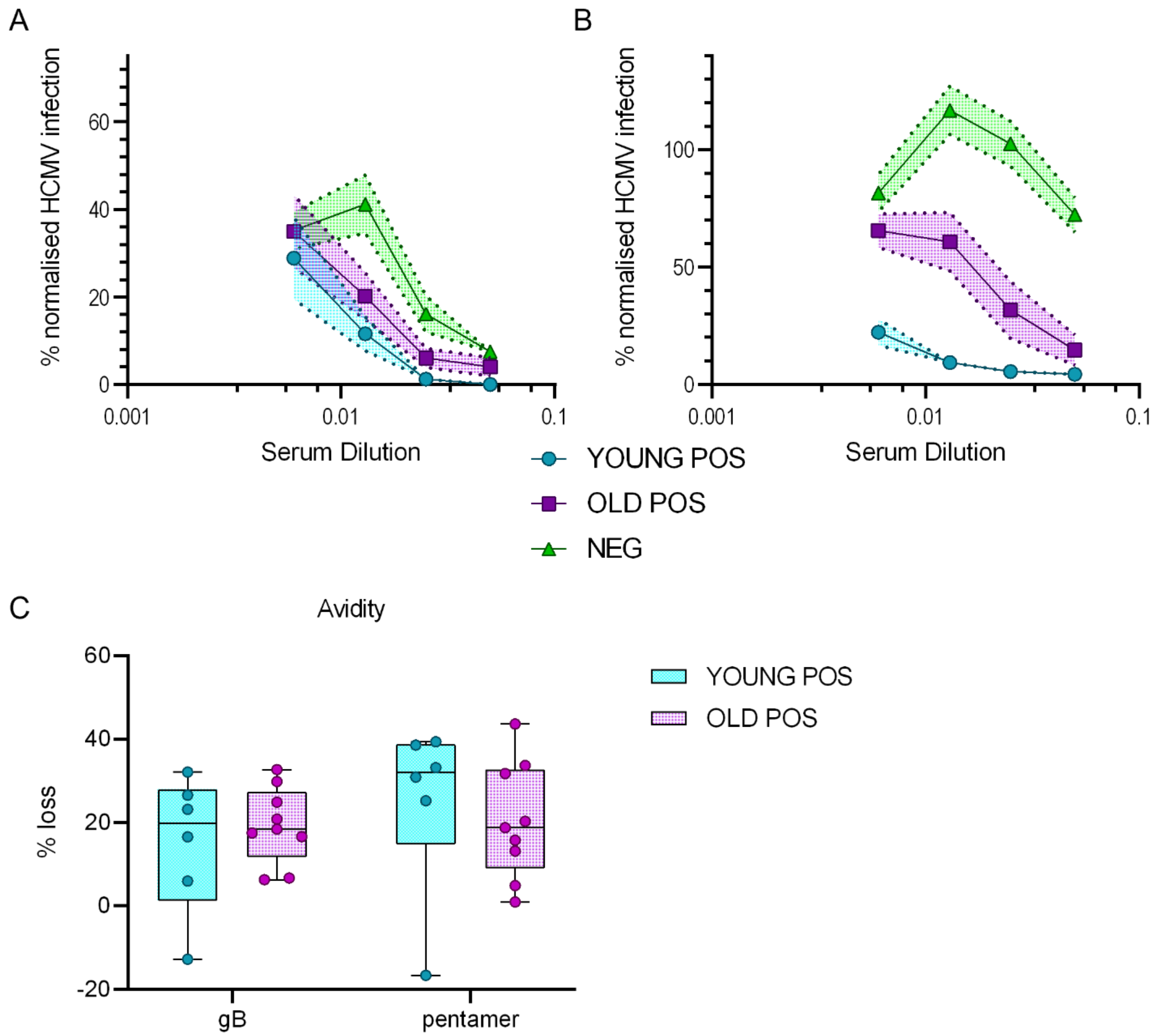

Supplementary Figure S11 – Neutralisation activity of all donor cohorts and Avidity of serum HCMV gB and pentamer specific IgG interactions

Neutralization assays with heat inactivated sera were performed on fibroblasts (A) and epithelial cells (B), serum was diluted and pre-incubated with the fluorescence tagged virus prior to adding to the cells. The mean +/- standard error of the mean of the normalized viral infection for the three cohorts analysed, young positive (turquoise points and shading), old positive (purple points and shading) and seronegative donors (green points and shading) for late (GFP+ and mCherry+) gene expression for both cell types are shown. The avidity of the interaction of serum IgG specific to HCMV gB and pentameric complex (gH/gL/pUL128-130-131 (5)) proteins in young and old HCMV seropositive donors was measured by the addition of a urea treatment to displace weakly bound antibody. 96 well ELISA plates were coated overnight at 4°C with either gB protein (Abcam, Cambridge, UK) or Pentamer protein (Native Antigen Company, Oxford, UK) and then blocked (2% FCS in PBS) for 1 hour at room temperature. Pre-diluted serum samples from the AQUARIA donor cohort were added in duplicate for each condition alongside relevant sample controls and incubated for 1 hour at room

temperature. Urea treated wells then had the serum samples removed and the 6M urea (Thermo Fisher Scientific) in PBS wash added to the wells for 8 minutes, followed by 3 subsequent wash steps using the urea wash buffer. The untreated wells were then washed with normal wash buffer (0.1% tween in PBS), All wells were incubated with 1:10 000 dilution of anti-human HRP conjugated antibody (goat-anti-human IgG, Dianova via Stratech, Ely, UK) and incubated for 1 hour at room temperature and then washed. After washing, tetramethylbenzidine peroxidase substrate was added to each well for 30 minutes, diluted 1:1 in peroxidase substrate solution B (KPL, USA). The reaction was stopped by adding 100 µl of 1M phosphoric acid to each well. The optical density at 450 nm (OD450) was determined using an Emaxmicroplate reader (Eurofins MWG Operon). The percentage loss of antibody was determined by the following equation:

$$\% \, loss = (\text{mean OD of urea-treated sera} \div \text{mean OD urea-untreated sera}) \times 100$$

The results of this calculation are shown as a box and whisker minimum to maximum plot (with median and upper and lower quartiles represented by the box) (C) for each protein with the young HCMV positive donors in turquoise and the old HCMV positive donors in pink shading and points.

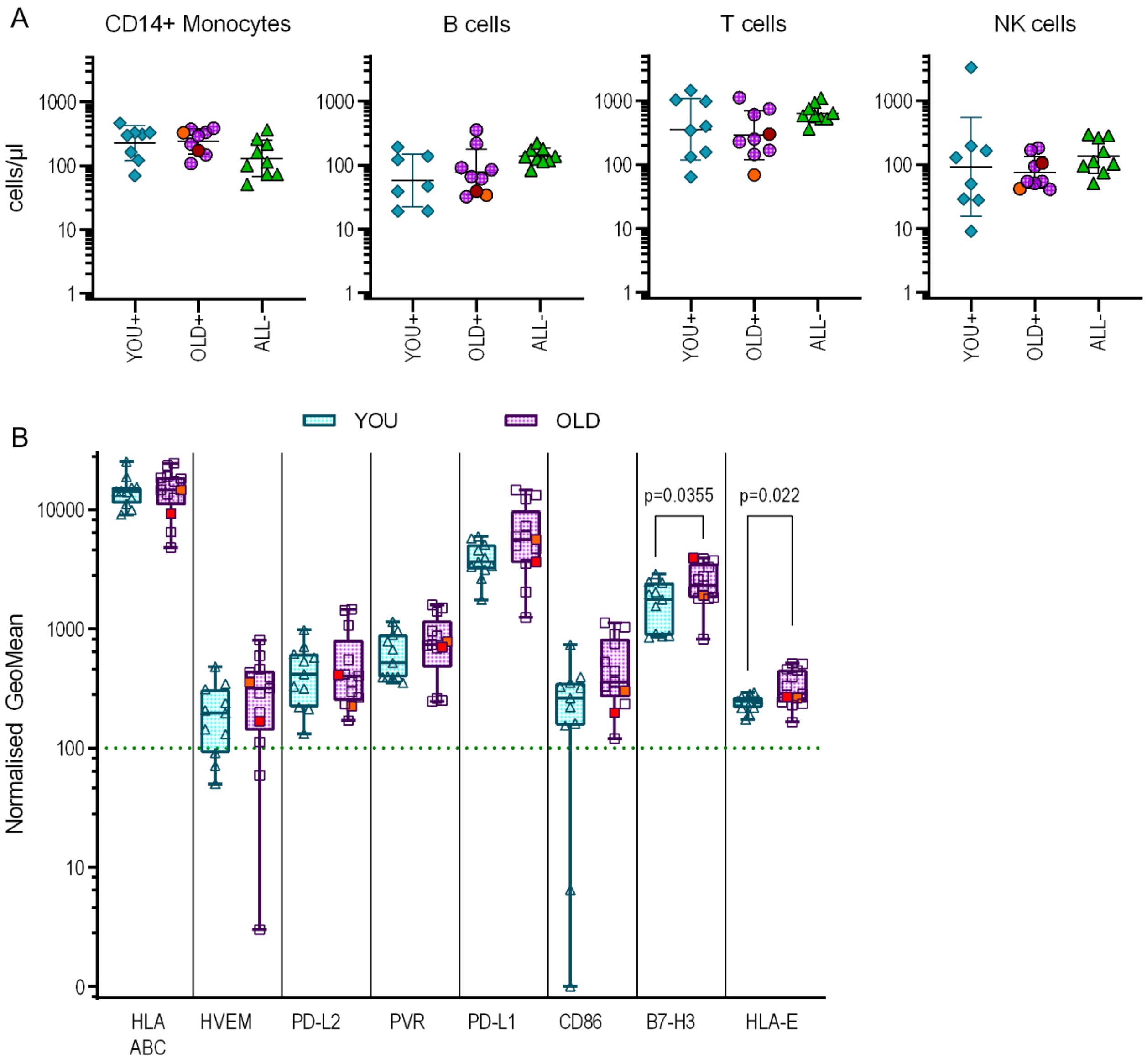

Supplementary Figure S12 – Identification of Saliva HCMV DNA+ donors in absolute count and Fibroblast inhibitory ligand molecules

Absolute count data for the Monocytes, B cells, T cells and NK cells according to the young and old seropositive and seronegative groups are shown with donors AQU007 (orange points) and AQU022 (red points) highlighted (geomean and geometric standard deviation shown) (A). Dermal fibroblast inhibitory ligand expression by young and old groups as box and whisker min – max plots are shown with donors AQU007 (orange points) and AQU022 (red points) highlighted (B).

3 References

1. Philippeos C, Telerman SB, Oules B, Pisco AO, Shaw TJ, Elgueta R, et al. Spatial and Single-Cell Transcriptional Profiling Identifies Functionally Distinct Human Dermal Fibroblast Subpopulations. *J Invest Dermatol* (2018) 138(4):811-25. Epub 2018/02/03. doi: 10.1016/j.jid.2018.01.016.

2. Pangrazzi L, Reidla J, Carmona Arana JA, Naismith E, Miggitsch C, Meryk A, et al. Cd28 and Cd57 Define Four Populations with Distinct Phenotypic Properties within Human Cd8(+) T Cells. *Eur J Immunol* (2019). Epub 2019/11/23. doi: 10.1002/eji.201948362.

3. Houldcroft CJ, Jackson SE, Lim EY, Sedikides GX, Davies EL, Atkinson C, et al. Assessing Anti-Hcmv Cell Mediated Immune Responses in Transplant Recipients and Healthy Controls Using a Novel Functional Assay. *Front Cell Infect Microbiol* (2020) 10. doi: 10.3389/fcimb.2020.00275.

4. Forrester MA, Wassall HJ, Hall LS, Cao H, Wilson HM, Barker RN, et al. Similarities and Differences in Surface Receptor Expression by Thp-1 Monocytes and Differentiated Macrophages Polarized Using Seven Different Conditioning Regimens. *Cell Immunol* (2018) 332:58-76. Epub 2018/08/06. doi: 10.1016/j.cellimm.2018.07.008.

5. Lilleri D, Kabanova A, Lanzavecchia A, Gerna G. Antibodies against Neutralization Epitopes of Human Cytomegalovirus Gh/Gl/Pul128-130-131 Complex and Virus Spreading May Correlate with Virus Control in Vivo. *J Clin Immunol* (2012) 32(6):1324-31. Epub 20120727. doi: 10.1007/s10875-012-9739-3.
